# How to Train a Cell–Cutting-Edge Molecular Tools

**DOI:** 10.3389/fchem.2017.00012

**Published:** 2017-03-10

**Authors:** Jakub Czapiński, Michał Kiełbus, Joanna Kałafut, Michał Kos, Andrzej Stepulak, Adolfo Rivero-Müller

**Affiliations:** ^1^Department of Biochemistry and Molecular Biology, Medical University of LublinLublin, Poland; ^2^Postgraduate School of Molecular Medicine, Medical University of WarsawWarsaw, Poland; ^3^Turku Centre for Biotechnology, University of Turku and Åbo Akademi UniversityTurku, Finland; ^4^Department of Biosciences, Åbo Akademi UniversityTurku, Finland

**Keywords:** protein interactions, signaling pathways, cell communication, optogenetics, gene editing, synthetic biology, gene expression regulation, controlling behavior

## Abstract

In biological systems, the formation of molecular complexes is the currency for all cellular processes. Traditionally, functional experimentation was targeted to single molecular players in order to understand its effects in a cell or animal phenotype. In the last few years, we have been experiencing rapid progress in the development of ground-breaking molecular biology tools that affect the metabolic, structural, morphological, and (epi)genetic instructions of cells by chemical, optical (optogenetic) and mechanical inputs. Such precise dissection of cellular processes is not only essential for a better understanding of biological systems, but will also allow us to better diagnose and fix common dysfunctions. Here, we present several of these emerging and innovative techniques by providing the reader with elegant examples on how these tools have been implemented in cells, and, in some cases, organisms, to unravel molecular processes in minute detail. We also discuss their advantages and disadvantages with particular focus on their translation to multicellular organisms for *in vivo* spatiotemporal regulation. We envision that further developments of these tools will not only help solve the processes of life, but will give rise to novel clinical and industrial applications.

## Introduction

**Figure 1:**
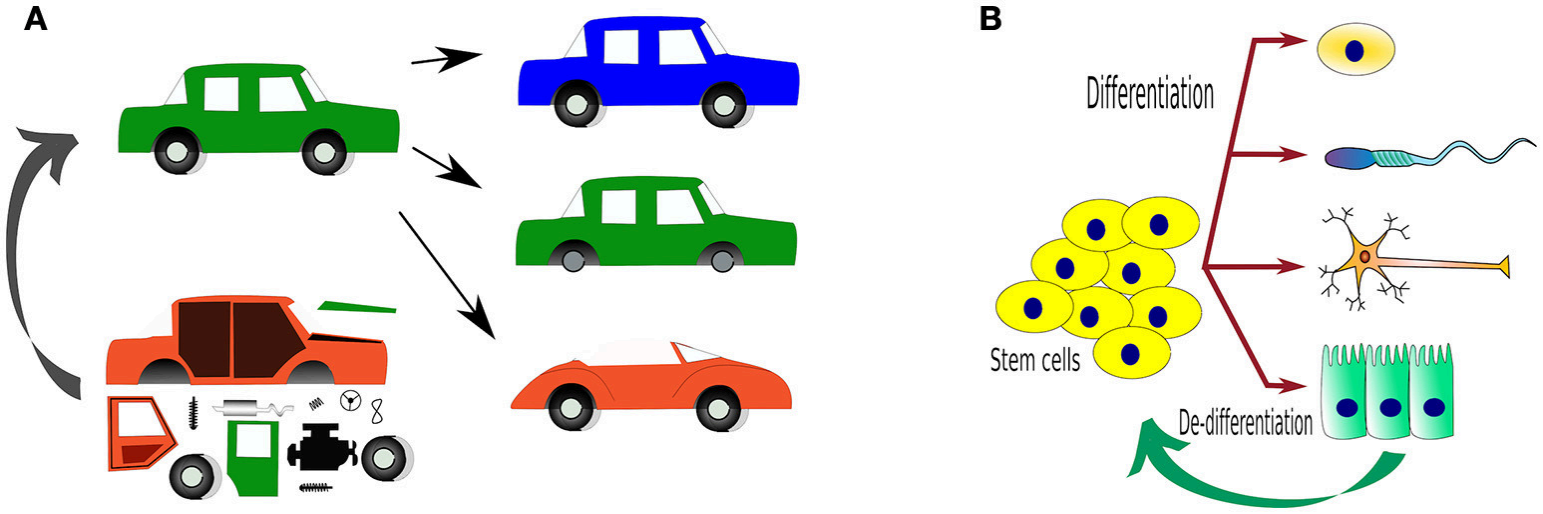
**Traditional ways to study organisms, either by introducing or deleting genes and *expecting* phenotypical changes. (A)** An analogy using a car as a “complex” system, where changes in the components of the car by adding (transgenic, TG), deleting (KO) or replacing/repairing (KI) “parts” (representing genes). Can we one day use parts to create a whole?; **(B)** The main reason why we would like to control living systems is to control how one cell behaves and in this way, determine what it does e.g. differentiation. The tools to achieve such fates are only beginning to come of age.

For millennia, our species has tried to control the environment around us to facilitate our activities. This has led to technological inventions from housing to space-probes that (crash) land on a different planet. Yet, when it comes to living organisms, our control over their behaviors has only been partial. Initial works have been done using small molecules to activate or inhibit (hopefully) single cellular functions. Later on, gene augmentation or elimination has been the focus of much of cell biology, as well as transgenic and knockout (KO) models, for the last two decades. With this, we have attempted to understand what occurs when a gene is inhibited/removed, or if we can influence the phenotype of cells or organisms by adding or exchanging genes (knock-in, KI) (Figure 1).

Although, these experiments have produced a trove of valuable data, it can be considered as just the first steps to real control of phenotypical changes. A novel branch of molecular biology, called synthetic biology, has stepped in by providing a suite of innovative tools that enable an unprecedented control of cellular processes, and eventually organisms. Synthetic biology mainly involves the rational design and engineering of novel biological devises, or systems, by coupling different biological parts or modules (Kelwick et al., 2014).

In this review, some of the most innovative tools for the detailed manipulation of cellular processes are presented, discussing their advantages and disadvantages, as well as their potential translational application to multicellular organisms which would be the final goal.

## Right to assemble

Cells systematically use protein assembly or dissociation as cues to perform the most complex of biological functions. Therefore, the manipulation of protein-protein interactions is essential for the development of any cell controlling system.

Since proteins are the *workhorses* of cells, these have been the main focus of most of the research involving control of cellular behavior. Proteins interact with each other, as well as with other components of the cell, which is done in an incredibly active manner. Virtually all cellular processes involve the formation of protein complexes, frequently involving RNA, DNA, and/or other biological molecules, too.

### Location, location, location

One of the most important factors affecting protein function is localization. Where the protein locates determines its interaction partners and thus its functions. Cells sort proteins using a series of encoded *signal peptides* (or *localization signals*) within the protein, analogous to postcodes, that determine where the protein should be transported e.g., to the nucleus, to mitochondria, to the cell membrane, or to be secreted. Signal peptides were first described by Günter Blobel and Bernhard Dobberstein in a landmark paper (Blobel and Dobberstein, 1975) that eventually lead to the Nobel prize in Physiology or Medicine for Blobel in 1999.

The modification of these signals results in changes in the localisation of proteins and hereby their functions. This can be used to our advantage, for example localization signals can be added to exogenous proteins (usually encoded in a plasmid) so their localisation is predetermined. Adding a nuclear localisation signal (NLS) to virtually any protein would result in protein re-routing to the cell nucleus. The reverse process is also possible, using nuclear export signal (NES) to export a protein out of the nucleus. Having both NLS/NES will result in the protein being shuttled between cytoplasm and the nucleus.

Likewise, mitochondrial targeting signals (MTS) have been used to attach proteins to the mitochondria's outer membrane for many different reasons, e.g., to label mitochondria by use of fluorescent proteins (FPs) or sequestering proteins to the mitochondria membrane (see below).

Experimental swapping of localization signals are impressive tools to study protein function as shown in the following study. Cytoskeleton remodeling proteins Ena (Mammalian Enabled Homolog) and VASP (Vasodilator-Stimulated Phosphoprotein) are cytosolic proteins often found in focal adhesions and leading edge of fibroblasts, and so assumed to play crucial roles in remodeling of the actin cytoskeleton that promote cell motility. Using localisation signals, Bear and collaborators studied Ena or VASP by either sequestering onto the mitochondrial outer membrane or directing them to the cell membrane, thus to the vicinity of focal adhesions, of fibroblasts. Against all expectations, Ena/VASP sequestration resulted in cytoskeleton remodeling and increased cell motility, whereas constitutive cell membrane localization reduced motility (Bear et al., 2000). This study highlights the importance of detailed experimentation at the molecular level to unravel cellular functions.

Localization can be used to bring proteins together or to separate them. For example, we found an extremely rare mutation in the gene that codes for a secreted protein, the luteinising hormone beta (LHB), in a patient. To prove that the wildtype (WT) and mutant proteins were expressed at the same level, but only the mutant was intracellularly retained, we fused the LHB to mCherry fluorescent protein, for tracking, and to (NLS)AmCyan via a 2A peptide. The 2A peptide is a self-cleaving peptide the separates the two proteins assuring both are expressed at equal concentrations. While the WT LHB-mCherry was normally secreted, and only visible inside secretion vesicles, the mutant was intracellularly retained. Nuclear-AmCyan was then used to quantify for equal expression (Potorac et al., 2016).

A drawback of localization signals is their uncontrollability. Therefore, the next section is about additional modifications used to manipulate protein localization.

The discovery that small compounds can either induce the dimerization or stabilization of some proteins, has prompted their use to regulate protein-protein interactions or protein levels in the cell, respectively.

### The chemistry between us

**Figure 2:**
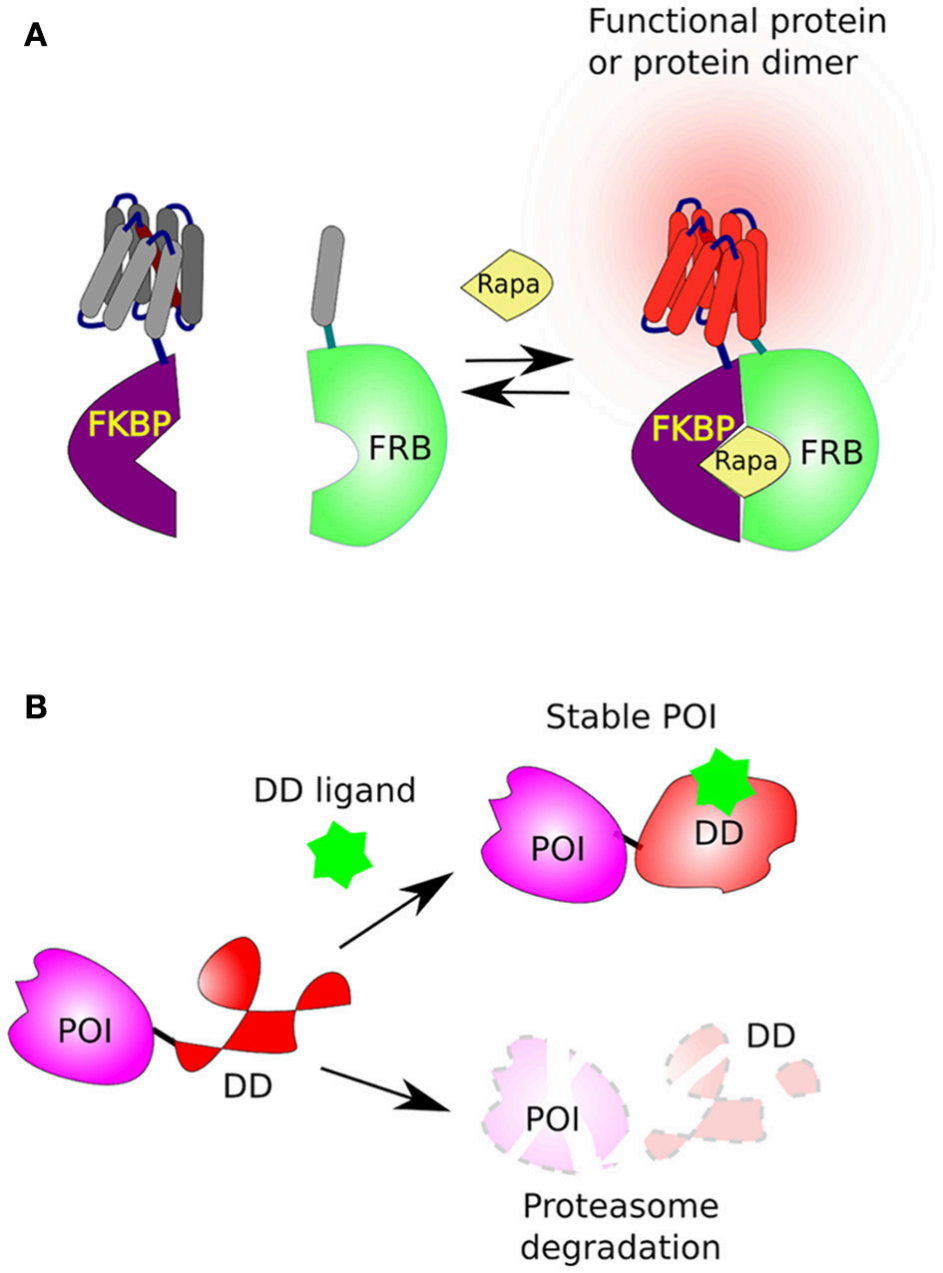
**Chemical-induced dimerization and protein stabilization. (A)** The FKBP/FRB system based on rapamycin-induced protein dimerization. This system brings two protein, or two complementing fragments, together, when one is fused to FKBP and the second to FRB in the presence of rapamycin (Rapa). **(B)** The fusion of a destabilizing domain (DD) to a protein of interest (POI) leads to its rapid degradation by the proteasome. However, in the presence of a suitable ligand [Shield1 for FKBP or Trimethoprim (TMP) for DHFR] the DD is stabilized and the POI accumulates in the cell.

One of the best well-described methods where a small compound triggers protein dimerization involves rapamycin, a macrolide antifungal antibiotic from *Streptomyces hygroscopicus*. Originally, rapamycin was found to form a functional complex with the FKBP protein (FK506-binding protein, aka FKBP12), a complex that specifically binds, and inhibits, the mammalian TOR complex 1 (mTORC1). The mTOR's domain, which directly interacts with rapamycin-FKBP moiety, was named FRB (FKBP–rapamycin binding domain of mTOR) (Chung et al., 1992; Sigal and Dumont, 1992). The reciprocal affinity of these two rapamycin-binding domains (FKBP and FRB) has been underpinned for the development of chemical-inducible dimerization (CID) (Banaszynski et al., 2005), where two proteins of interest (POI) or two complementing fragments of a protein, are each fused to either FKBP or FRB and thereby can be united by addition of rapamycin (Figure 2A). Below we describe a couple of well-designed applications on how CID has been used to understand and control cellular functions.

During the last checkpoint in mitosis, M or spindle checkpoint, one of the most sophisticated protein complexes in the cell is meticulously assembled. The complex ensures that the sister chromatids are aligned and attached to the microtubule spindles via kinetochores. Once kinetochores are correctly attached to the spindle, the checkpoint is inactivated by protein dissociation and cell division can furthermore proceed. In order to determine which of the checkpoint proteins are able to reactivate the checkpoint, Ballister, Riegman, and Lampson used CID to temporarily re-localize checkpoint proteins to the kinetochore. They fused the mitotic association protein Mis12, a component of the kinetochore, with FKBP and green FP (GFP), and FRB was fused to mCherry and checkpoint Mad1 protein. In the presence of rapamycin, Mad1 was recruited into the kinetochore, as visualized by GFP/mCherry, triggering the reactivation of the mitotic checkpoint to confirm that Mad1 is a crucial checkpoint component (Ballister et al., 2014; Ballister and Lampson, 2016).

Fusing FRB and FKBP domains, surprisingly, does not result in a molecular *clamp* in the presence of rapamycin, instead it forms FRB-FKBP tetramers: where the FKBP of one moiety dimerises with the FRB of another, occurring thrice more, the last FKBP binds to the first FRB. The cellular *gatekeeper* p53 plays essential roles in cycle arrest, senescence, apoptosis, and DNA damage repair by regulation of the expression of hundreds of genes. A tetrameric conformation of p53 is required to bind to target DNA, which is normally achieved via a tetrameric domain. Therefore, the DNA binding domain of p53 was fused to FRB-FKBP. Upon addition of rapamycin, the engineered p53 tetramerised and activated target genes—an inducible p53 for detailed studies in p53's tumor suppression functions (Inobe et al., 2015).

The FKBP/FRB dimerization has been widely used, more in-depth examples can also be found in the following reviews (Putyrski and Schultz, 2012; DeRose et al., 2013; Feng and Arnold, 2016). A drawback in the use of rapamycin is that it targets mTOR which is a master regulator of cell growth and metabolism (Li et al., 2014; Fischer et al., 2016).

Exploiting CID plus delocalization, Robinson and colleagues generated a technique they named *knocksideways* (a British expression meaning “to take by surprise”) where FRB is anchored to the outer membrane of the mitochondria via a mitochondria targeting signal (MTS). FRB-MTS functions as a *trap* for FKBP-fused proteins in the presence of rapamycin—sequestering proteins away from their site of action. Using *knocksideways* the authors sequestered the adaptor protein 2 (AP-2), normally recruited to endocytotic vesicles, and demonstrated that clathrin-mediated endocytosis of transferrin requires AP-2 (Robinson et al., 2010). *Knocksideways* is able to control a cellular process by temporarily sequestering key molecular components.

While this procedure is fast and allows immediate removal of molecular players, avoiding compensations common in long knockdown (silencing) or knockout (KO) experiments, it requires the removal, by silencing or KO, of the endogenous POI, or more sophisticatedly a knock-in (KI), inserting *FKBP* as a fusion tag to the endogenous gene of interest. Although this domain could be easily adapted to exogenous proteins in synthetic biology, the side effects of rapamycin are an issue nevertheless.

### An act of disappearance and reappearance

Protein turnover is determined by its degradation rate—mostly performed by the proteasome. Misfolded or aged proteins are labeled with a small protein tag called ubiquitin by ubiquitin ligase, then such ubiquitinated proteins are mostly routed to the proteasome for rapid degradation (Hershko et al., 1980).

The first system based on the control of protein stability was reported in the middle of the 90s. A mutant of mammalian dihydrofolate reductase bearing an *N*-terminal arginine (Arg-DHFR), was shown to be unstable at 37°C but stable at lower temperatures (~23°C) in yeast (Dohmen et al., 1994; Lévy et al., 1999). Interestingly, addition of the DHFR inhibitor, methotrexate, partially protected Arg-DHFR from degradation (Lévy et al., 1999), which was suggested as a potential method to control protein degradation.

Subsequently, the laboratory of Thomas Wandless developed two destabilization domains (DDs), that are rapidly destabilized and degraded by the proteasome. The first is a 107 residues long (12-kDa) FKBP derivative (DD-FKBP) which is stabilized by a ligand, named morpholino-containing ligand (Shield-1) (Banaszynski et al., 2006; Haugwitz et al., 2008). The second was based on the Arg-DHFR, but instead, using prokaryotic *E. coli* dihydrofolate reductase (ecDHFR) mutants that were engineered to be unstable in the absence, but stabilized in the presence, of the cell-permeable prokaryotic-DHFR inhibitor trimethoprim (TMP) (Iwamoto et al., 2010; Figure 2B).

DD-FKBP/Shield-1 and DD-ecDHFR/TMP have been used in living cells and animal models (An et al., 2015) including the accumulation of a reporter DD-yellow fluorescent protein (DD-YFP) in the brain of rats, transduced by a lentivirus, after TMP was administrated in drinking water (Iwamoto et al., 2010; Tai et al., 2012). Moreover, with the use of DD-ecDHFR/TMP, Quintino and collaborators were able to control the level of glial cells-derived neurotrophic factor (GDNF), a protein that exerts neurotrophic and neuroprotective effects on dopamine neurons, in a rodent model of Parkinson's disease (Quintino et al., 2013) confirming the great therapeutic potential of GDNF against Parkinson's disease.

TMP has several advantages: it acts on prokaryotic ecDHFR with virtually no effect on mammalian DHFR, has low toxicity, and is able to cross the blood-brain barrier. Moreover, these two systems (DD-FKBP/Shield-1 and ecDHFR/TMP) can be used orthogonally, and both inhibitors do not seem to have any major side effects in animal models. The cell specificity *in vivo* could be achieved by using tissue-specific promoters; further, protein accumulation is reversible. Conversely, DDs are large proteins that can cause secondary structural effects to the fused protein, the accumulation kinetics will be strongly influenced by gene expression and inhibitor doses, and they cannot be regulated at the subcellular level.

All abovementioned systems are based in the stabilization of domains that otherwise destine the fusion protein for degradation. Can a protein be removed at will? The auxin-inducible degron (AID) was originally discovered in plants (Nishimura et al., 2009) and subsequently optimized for mammalian cells (Fallis, 2009; Morawska and Ulrich, 2013). Auxins, such as indole-3-acetic acid (IAA), function as hormones regulating gene expression in plants (Fallis, 2009). Proteins having an AID tag are normally expressed, yet, in the presence of IAA, they interact with F-box protein TIR1, an interaction that triggers ubiquitination by E3 ligase and consequently proteasomal degradation (Morawska and Ulrich, 2013) in 0,5–2 h (Zhang et al., 2015; Wood et al., 2016). AID has also been successfully adapted to the nematode *Caenorhabditis elegans, where the main component of AID*, TIR1, was expressed under the control of different promoters to drive tissue- and stage-specific expression. While the AID tag was introduced into endogenous nuclear hormone receptors *nhr-23* and *nhr-25* genes, as well as meiosis-specific gene *dhc-1* (dynein heavy chain) using CRISPR/Cas9 technology (see below). Upon addition of auxin both of the nuclear receptors were dynamically removed, demonstrating that the decrease of NHR-25 receptor produced larval arrest, molting defects and gonads abnormalities, whereas auxin-induced NHR-23 depletion was associated with larval arrest only. Auxin-induced degradation of DHC-1 protein exerted defects in chromosome synapsis, included global disorganization of germline nuclei and defects in oocyte maturation in *Caenorhabditis*, which proves its crucial role in meiosis (Zhang et al., 2015).

Ongoing efforts to develop more potent auxin agonists are on the way, although the properties of IAA (water solubility, size, low toxicity, and a low cost) may prove difficult to improve upon. The need of small molecules in all chemical-induced systems, produces a series of challenges for translation to *in vivo*. These small molecules should not interact with other cellular components, have minimal-to-none toxicity and immunogenicity, and should be delivered to all tissues, or, in some cases, to specific tissues. An additional challenge for most small molecules is to pass through the blood-brain or blood-testis barriers.

## The first-light on optogenetics

**Figure 3:**
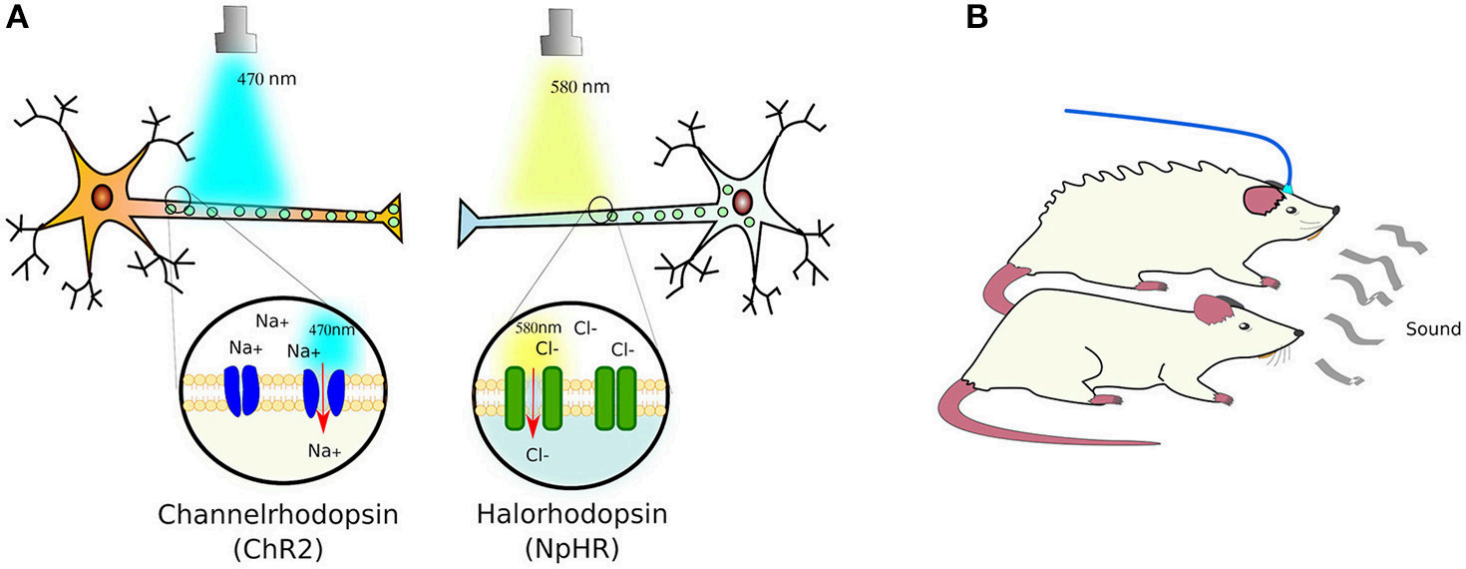
**Light-sensing ion pumps. (A)** The photosensitive channelrhodopsin-2 (ChR2) and halorhodopsin (NpHR) ion channels expressed in neurons allow the polarization of the cells by sodium (Na^+^) or chloride (Cl^−^) influx. **(B)** Using these light-activated rhodopsins, it has been possible to control the responses of animal models to e.g., elicit defensive behaviors in mice (see text for details).

Expectedly, plants, algae and bacteria have proteins that respond to light. Indeed, even animals have proteins that respond to light—such as the opsin receptors. Although, in animal opsins receptors are G protein-coupled receptors (GPCRs) while in microorganisms opsin receptors are ion channels instead. The pioneer of these ion receptors, cloned from *Chlamydomonas reinhardtii*, was channelrhodopsin-2 (ChR2). This cation channel was customized to mammalian cells, proving its functionality by transfecting neurons and exposing them to blue-light (470 nm), which induced polarization by admitting sodium in Nagel et al. (2003) and Boyden et al. (2005). This was followed by the *Natronomonas pharaonis* halorhodopsin (NpHR) chloride pump (Zhang et al., 2007), a yellow-light sensing receptor (589 nm), which allows chlorine ions into cells, enabling to turn firing neurons off (Figure 3A).

The immediate success of these receptors, prompted a search in plants, fungi, bacteria and algae for novel proteins that are able to react to light. What has been found so far is a fascinating collection of proteins that change function upon light activation. Due to the characteristics of light, these light-sensing proteins respond to different wavelengths, some of them only to short wavelength windows while others respond distinctively to different light hues.

As a proof of concept, ChR2, NpHR, and a new red-light (566 nm) activated protein pump, Arch (archaerhodopsin; Chow et al., 2010), were introduced into the brains of mice using Cre-conditional adeno-associated viral vectors, in which Cre recombinase (see Box 3) expression was controlled by neuron-type specific promoters: *parvalbumin* for expression in GABAergic interneurons, and *somatostatin* for expression in basolateral amygdala principal neurons. Only the parvalbumin- or somatostatin-positive cells developed light sensitivity to the correct wavelength. In this manner, the authors managed to control the activation of fear memory without a conditioned stimulus—in this case, a sound associated with a footshock—or inhibited fear responses upon auditory stimulation (Wolff et al., 2014). In a separate study, the photosensitive ChR2 and NpHR ion channels were expressed in the cortical amygdala neurons of mice under the control of the *arc* promoter—specific to these neurons. Activation of the cortical amygdala neurons by odors secreted by predators, such as TMT (2,4,5 dihydro 2,5 trimethylthiazoline), produce defensive behavior in mice. Light-activation of NpHR silenced the olfactory bulb and suppressed the aversion to TMT, while ChR2 activation induced the defensive response in the absence of TMT (Figure 3B). With this, the authors demonstrated that, by optogenetical affecting the neural circuit that transmits information from the olfactory bulb to cortical amygdala, they can control the innate behavior of animals (Root et al., 2014).

Neurons are not alone to respond to an influx of ions, muscle cells respond by contracting upon sodium influx. Based on this, Park et al. layered ChR2-expressing cardiomyocytes in a serpentine pattern on a ray fish-shaped elastomer with a gold skeleton—to retract to the original shape, in an attempt to create prototypes for organ bioengineering. Photoactivation triggered sequential muscular contractions, creating undulatory phototactic locomotion of this biorobot (it swam in direction to light!) (Park et al., 2016).

Since red-light has a better tissue penetrance, is less absorbed by blood, and does not appear to interfere with normal visual function, a green-light-responding channelrhodopsin from *Volvox carteri* has been engineered to respond to orange-to-red wavelengths (590–630 nm) (Lin et al., 2013). This red-activatable Channelrhodopsin (ReaChR), when expressed in gustatory neurons, was used for the precise regulation of male courtship song of freely moving adult *Drosophila* flies, to discover a two neuronal-regulated command-like components (Inagaki et al., 2014).

For more detailed focused reviews, refer to the following reviews (Rein and Deussing, 2012; Tischer and Weiner, 2014; Guru et al., 2015).

Other non-membranous optogenetic proteins shall be described below, following presentation of a different approach to rationally generate light-responding membrane receptors, now based on the structural similarities between mammalian light-sensing receptors (opsins) - GPCRs themselves - and other GPCRs.

### Light my pathway

#### Direct light-activation of GPCRs

**Figure 4:**
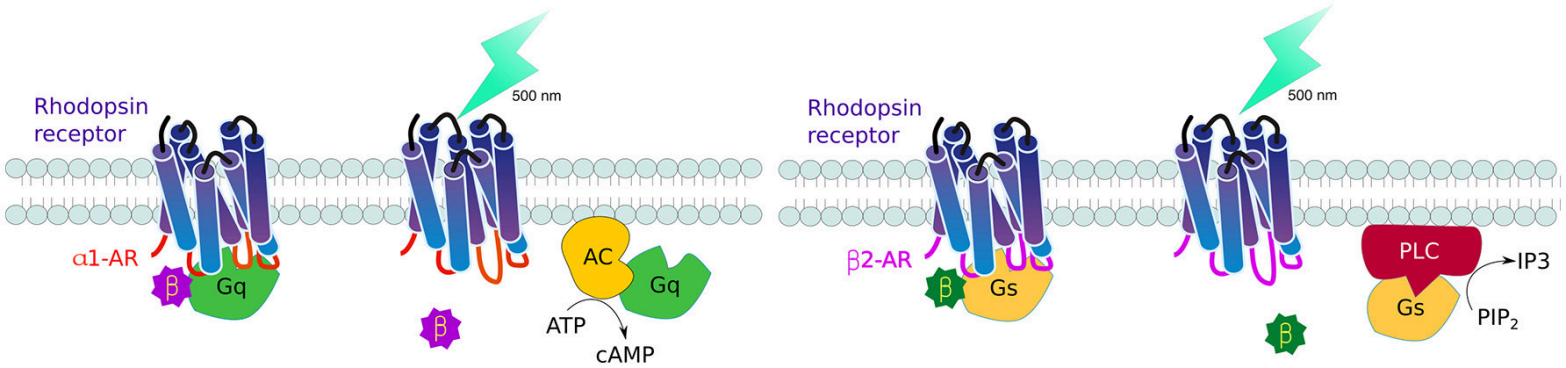
**Controlling GPCR signaling by photoactivation**. By interchanging the intracellular domains (loops) of the Gt-coupled bovine green-absorbing rhodopsin receptor for either the Gq-coupled human alpha 1 adrenogenic receptor (α1AR) (red), or Gs-coupled hamster beta 1 adrenogenic receptor (β2AR) (violet), it is possible to activate single downstream pathways upon light activation: opto-α1AR activates adenylate cyclase and cyclic adenosine monophosphate (cAMP) production while opto-β2AR activates phospholipase C leads to increase inositol triphosphate (IP_3_).

GPCRs, all 7 serpentine transmembrane receptors that take a barrel-like conformation, are the largest family of receptors in vertebrates. Controlling these receptors is therefore an important area of interest to understand cell communication. The challenge was to generate receptors that are triggered by light, but transduce predetermined signaling pathways. To this end, Airan and collaborators rationally designed chimeric receptors where they kept the extracellular and transmembrane domains of the Gt-coupled bovine green-absorbing rhodopsin and exchanged the intracellular loops—reponsible for G protein coupling - to those of either Gq-coupled human alpha 1 (α1AR) or Gs-coupled hamster beta 1 (β2AR) adrenoceptors. The resulting receptors, named optoXRs, proved able to activate the expected intercellular pathway upon light activation: opto-α1AR activated Gq-responsive adenylate cyclase and downstream cyclic adenosine monophosphate (cAMP), while opto-β2AR activated Gs-activation of phospholipase C led to the second messenger inositol triphosphate (IP_3_) (Figure 4). These optoXRs only triggered biased-intracellular cascades, showing that endogenous modules can be incorporated into synthetic systems (Airan et al., 2009).

#### Secondary metabolites

Cyclic AMP (cAMP) is a common secondary metabolite downstream GPCR activation, which transduces intracellular signaling by activating kinases and ion channels. Therefore, cAMP control is an important cell communication signaling molecule. To date, two photo-activatable enzymes, one that produces and one that degrades cAMP, have been reported. The first is the microbial photoactivatable adenylyl cyclase (bPAC) from *Beggiatoa* which was found to increase 300-fold in cyclase activity (cAMP production) under 405 nm light, as compared to its non-stimulated state, in *E. coli, Xenopus* oocytes, and *Drosophila* neurons. In the latter it produced efficient light-induced depolarization and behavioral changes (Stierl et al., 2011). Light-inducible bPAC has also been used to rescue the function of rodent sperm that lack endogenous SACY (soluble adenylyl cyclase)—an essential cAMP-signaling component required for motility and capacitation of sperm (Jansen et al., 2015).

Degrading cAMP is as important as producing it for cell signaling responses, a task that is achieved by phosphodiesterases. With such aim, and with the use of *in silico* protein modeling, Gasser and collaborators noticed a strong structural similarity between the PDE domain of dimeric human phosphodiesterase 2A (PDE2A) and red-light responsive dimeric PhyB (see below) of *Deinococcus radiodurans*. By superimposing PhyB to PDE2A, they bioengineered a red-light-activating phosphodiesterase (LAPD), a chimera that hydrolyses up-to 6-fold more cAMP/cGMP upon light absorption in cell cultures or in zebrafish embryos. PhyB's photo-activated state can be reversed by far-red light (~700 nm), a feature that remains in LAPD. In addition, this red-light-PDE2A uses endogenous biliverdin as chromophore (Gasser et al., 2014), unlike PhyB which utilizes phycocyanobilin (see below).

Since bPAC and LAPD are activated by different wavelengths they can be used to fine-tune control of secondary metabolite signaling both *in vitro* and *in vivo*.

### Photosensing below the surface: non-membranous proteins

Abovementioned is that the discovery of optogenetic channels provoked a search for other proteins that respond to light, here are some of the best described.

Light oxygen voltage (LOV) is a small domain found in the *N*- or *C*-termini of some proteins from plants, fungi and some bacteria that responds to either of these three stimulations. There are two subclasses: modular LOV and short LOV (sLOV) (Crosson et al., 2003).

LOV domains were first identified in plant phototropins (LOV1 and LOV2) but also found conjugated to some regulatory proteins of circadian rhythms, phosphodiestrases, kinases, ubiquitin ligases, and DNA-binding proteins (Crosson et al., 2003). The first sLOVs identified were found in the YtvA protein of *Bacillus subtilis* (Losi et al., 2002). Homology sequence comparisons uncovered similar domains in proteins from different chemotropic and autotrophic prokaryotes (Jentzsch et al., 2009). Interestingly, the mechanism of action of LOV domain is conserved in prokaryotic and eukaryotic organisms (Jentzsch et al., 2009). LOV proteins have an important role in plant growth, development, regulation of circadian clock, stress response and adaptation, as well as in bacterial phototropism and cell-cell attachment (Lokhandwala et al., 2016).

The LOV domains found in many species were hypothesized to be responsible for sensing light, oxygen and/or voltage. Eventually, the *N*-terminal region of the *Arabidopsis*' nph1 was shown to contain a ~100 amino acid region highly conserved in many other sensor proteins (Huala et al., 1997). The LOV domain of nph1 from oat (*Avena sativa*) (AsLOV) was subsequently proven to be the photoactivatable domain of nph1 (Christie et al., 1999). The molecular mechanism involves photon absorption by a covalent bond between flavin cofactor (riboflavin, FMN or FAD) and a conserved cysteine residue, which remains stable for several seconds. This interaction leads to conformational changes and unwinding (undocking) of the *C*-terminal (Jα) helix (Wu et al., 2009).

**Figure 5:**
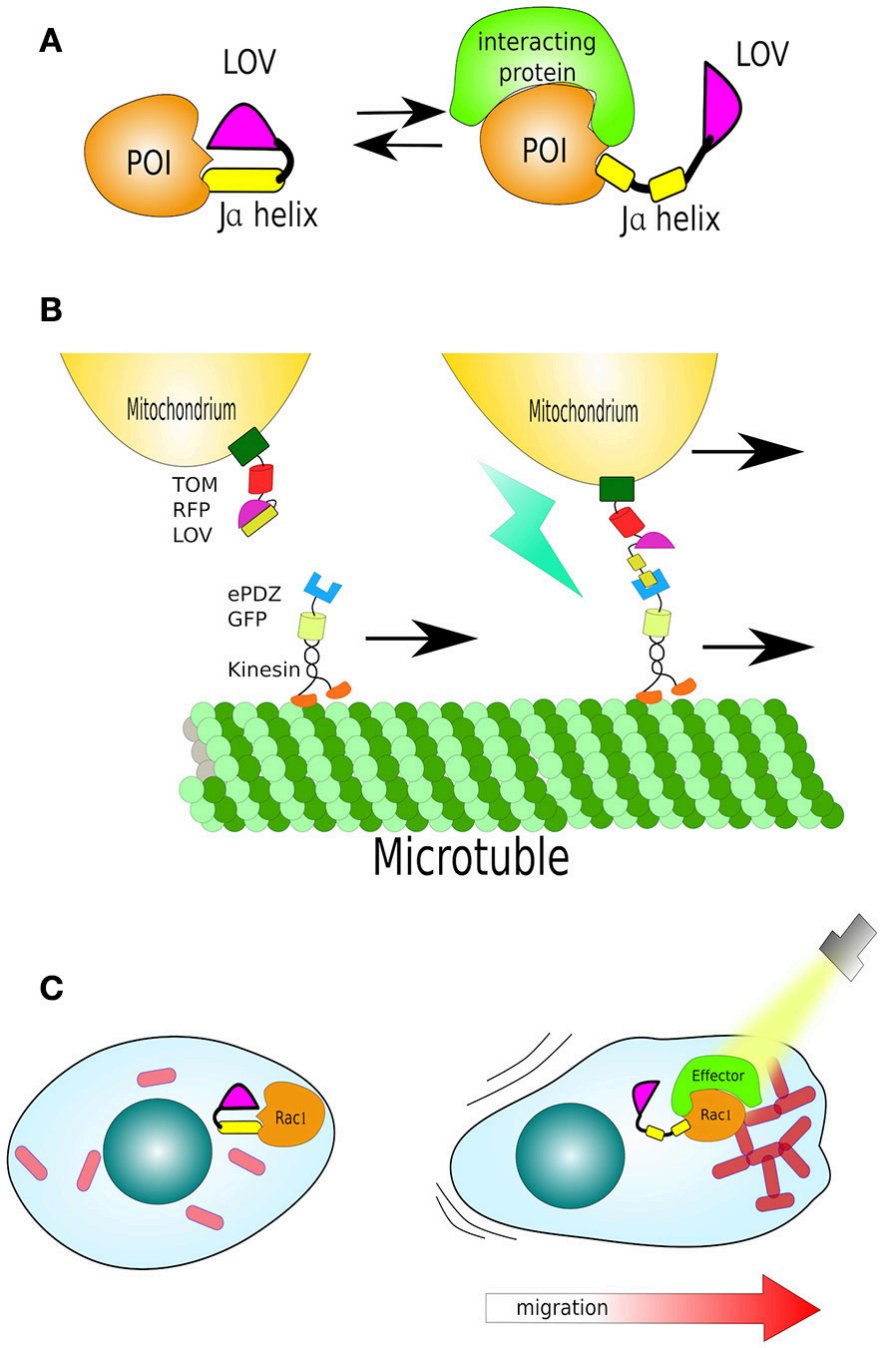
**Structure and function of LOV. (A)** The light/oxygen/voltage (LOV) domain is composed by a “core” and a helical domain called the Ja helix. Together they sandwich a flavin molecule in dark conditions. Upon light activation the Ja helix undocks. The LOV domain has been harnessed to cage signal peptides or functional domains of enzymes. **(B)** Organelle transport by LOV (mitochondria). A mitochondria-localized LOV domain caging a small peptide (LOVpep) that upon light exposure is exposed and bound by an engineered PDZ domain (ePDZ). By fusing ePDZ with motor protein kinesin, it is possible to control the translocation of mitochondria along microtubules in the axons of neurons. **(C)** Light-control on migration: In dark the LOV domain sterically blocks a constitutively-active Rac1. Uncaging Ja helix by light exposes Rac1 to its effector protein, triggering actin filament polymerisation and stimulated cell movement in the direction of the cell's illuminated edge.

In practice, LOV domains can be coupled to a protein of interest, usually in proximity to signal or functional domains, to conceal them by the folding structure of the LOV domain. Upon irradiation, the Jα helix is undocked and the signal or functional region exposed (Lokhandwala et al., 2016; Figure 5A). Combining a LOV domain adjacent to a localisation signal results in cytoplasmic localization, which upon light activation exposes the localization signal triggering re-localization (Yumerefendi et al., 2016). Several variants of this technique exist, such as LEXY, LINuS, LANS or LINX which consist of the concealment of nuclear export (NES) or/and nuclear localization (NLS) signals by LOV2, respectively (Niopek et al., 2014, 2016; Di Ventura and Kuhlman, 2016; Wehler et al., 2016; Yumerefendi et al., 2016). All of these modifications are short and genetically encoded domains that can be joined at the *C*- or *N*- terminus of proteins of interest (Wehler et al., 2016).

Beyond protein re-localization, although important as described above, the LOV domain has been adapted to the regulation of protein complexes, as well as the activity of enzymes. Here, there are some outstanding examples of these:

TULIPs (tuneable, light-controlled interacting protein tags) are two protein interaction systems based on the AsLOV2 synthetic domain caging a peptide epitope and, separately, its binding partner—a variant of the Erbin PDZ domain (ePDZ) (Strickland et al., 2012). PDZ domains mediate protein-protein interactions by binding to the *C*-terminus of their target protein, in a sequence-specific manner. The name PDZ is an acronym of the first three proteins in which these domains were discovered: PSD-95, DLG, and ZO-1 (Kennedy, 1995).

An engineered ePDZ domain, characterized for high-affinity and high-specificity to a peptide epitope (–SSADTWV–COOH) was selected (Skelton et al., 2003; Huang et al., 2009), while the peptide epitope was inserted into a truncated Jα helix *C*-terminal (called **LOVpep**) that had the lowest background activity—no binding to ePDZ in dark. After light induction, and Jα undocking, this additional peptide was rapidly bound by ePDZ domain, bringing the two proteins together.

By fusing GFP-LOVpep with transmembrane protein Mid2, thereby cell membrane localisation, the system was tested for its ability to recruit ePDZ-mCherry from cytoplasm to cell periphery. Most of the ePDZ-mCherry fusion was diffusely present in the cytoplasm in dark. After light excitation (473 nm), ePDZ-mCherry quickly colocalised with GFP-LOVpep at the cell membrane of yeast (Strickland et al., 2012). LOVpep/ePDZ dimerization has been also shown to operate in HeLa cells by either cell membrane or mitochondria colocalisation. Translocation is reversible even after 3 cycles of light excitation/recovery.

In yeast, mating behavior begins upon pheromone stimulation of a GPCR, which triggers two intracellular pathways: the MAPK pathway and the GTPase Cdc24 cascade. MAPK signaling leads to G1 arrest, and in consequence, growth inhibition, and is initiated by the recruitment of scaffold protein Ste5 and other components, such as Ste11, to the activated G protein. It is known that tethering Ste5 or Ste11 to the cell membrane activates the MAPK pathway (Winters et al., 2005). The Cdc24 cascade is required for polarized growth. The investigators then designed a TULIP for optical control of either MAPK or Cdc24 activation in budding yeast, without the involvement of G proteins or GPCRs by light-activation of the recruitment of ePDZ-Ste5delN (an allele deficient in G protein binding) or ePDZ-Ste11 (for MAPK activation), and Cdc24–ePDZb1 fusions to the membrane (Mid2-LOVpep, as above). As expected, all constructs showed no detectable dark-state changes, while growth arrest and/or polarization occurred upon continuous light excitation (Strickland et al., 2012). These results confirmed that this system can be successfully utilized for various purposes, including intracellular signaling.

van Bergeijk and colleagues used LOVpep not only to control protein movement but control organelle transport and positioning. Peroxisomes were labeled with LOVpep, fused to the peroxisome localization signal of PEX3. While ePDZ was fused with Kinesin-3, a plus-motor protein which moves along microtubules. Using monkey COS-7 cells as a model, the authors showed that blue irradiation triggered the translocation of peroxisomes from the cells' center to periphery, where most plus-microtubules are found. Furthermore, as the “cherry (not the fluorescent protein but the fruit of *Cerasus spp*) on the cake,” mitochondria-tagged by LOVpep, via mitochondrial membrane protein TOM20, were either translocated or anchored via microtubules along the axons of neurons, using ePDZ fused to Kinesin or SNPH, respectively (van Bergeijk et al., 2015; Figure 5B).

In another remarkable study, Wu et al. demonstrated that it is feasible to direct cell motility using LOV. They fused the LOV domain to the constitutively-active GTPase Rac1 (^*^Rac1), a regulator of actin cytoskeletal dynamics, and thus cell migration. In dark, the LOV domain sterically blocks ^*^Rac1, uncaging Ja helix by light leads disinhibition of ^*^Rac1, to bind its effector protein and polymerise actin filaments. This reversible mechanism was sufficient to create cell movement in the direction of light (Wu et al., 2009; Figure 5C).

Some of the systems described above could be considered *molecular machines*, as the definition for these is “an assembly of a distinct number of molecular components that are designed to perform machinelike movements (output) as a result of an appropriate external stimulation (input)” (Balzani et al., 2000). Most molecular machines combine synthetic with natural molecules to achieve certain activity e.g., movement. Until now, virtually all molecular machines have been tested using purified molecules under carefully controlled environments. An example of this: kinesin molecules used as nanocarriers as they move along purified microtubules attached to a surface (Bachand et al., 2005; Furuta et al., 2017). Molecular machines have the potential to harness the cells' metabolism as fuel, while driving pre-determined cellular activities. We refer the reader to specialized reviews on this subject (Balzani et al., 2000; Collin et al., 2001; Wesley and Browne, 2006; Feringa, 2007; Cheng and Stoddart, 2016).

### Photo-finish: light-induced dissociation

In all LOV stories (above), the storyline has been along the final union of two protein complexes and their cargoes. Yet, LOV has a dark side too, something that has recently given rise to LOVTRAP. LOVTRAP builds on the LOV2 domain anchored to the mitochondrial membrane, and the Zdk domain—a genetically engineered Z domain of bacterial protein A which has high affinity to the **dark state** of LOV2. Therefore, attaching Zdk to a POI results in colocalisation with mito-LOV2 (LOVTRAP). Cyan-light induces LOVTRAP to release Zdk-POI, a 150-fold change in the dissociation constant, to the cytosol, in a reversible manner. LOVTRAP has been successfully used to fine-tune the sequestration and release of several cytoplasmic proteins (GTP exchange factor Vav2, GTPase Rac1, and PI3K kinase) in mammalian cells, actions that modulated the activity of these proteins without any influence from endogenous regulatory pathways (Wang et al., 2016).

**Figure 6:**
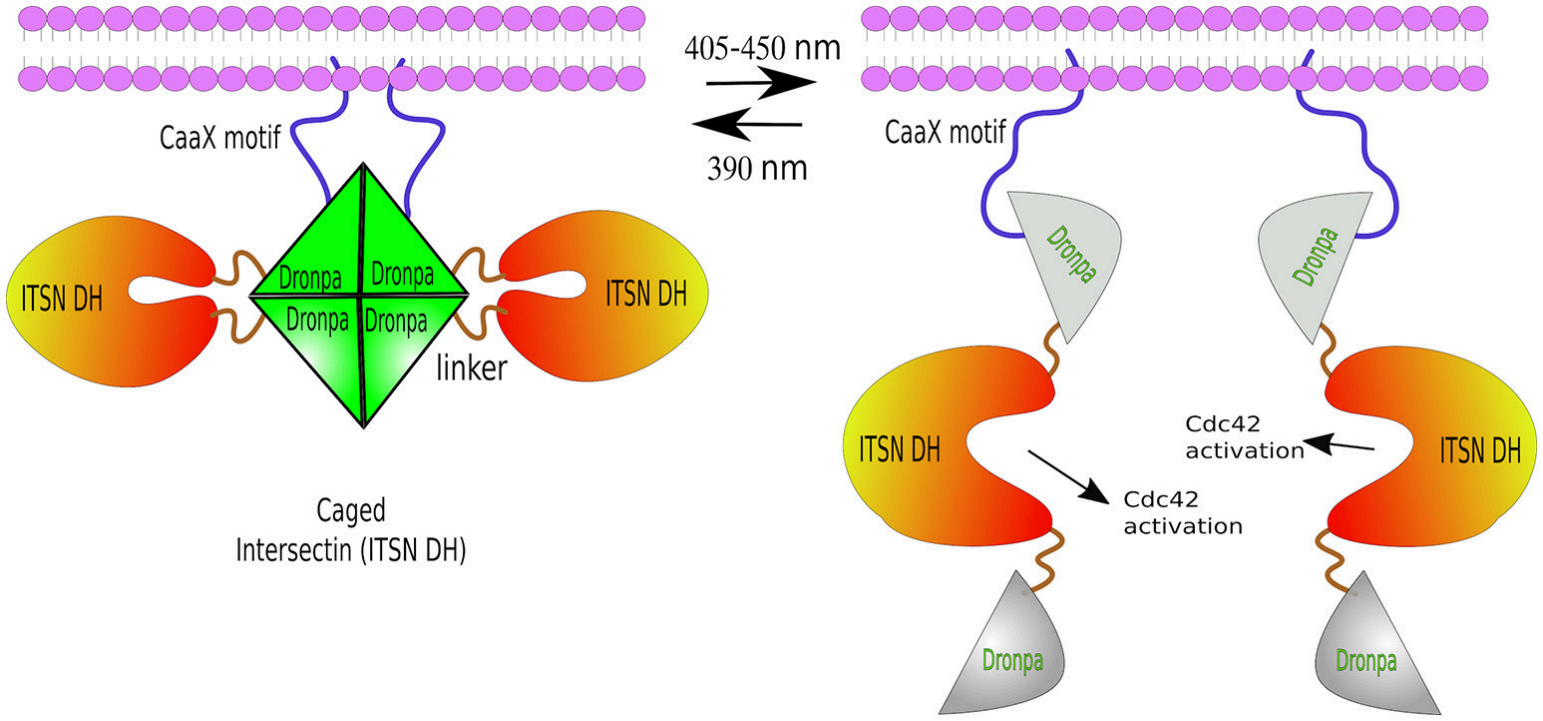
**Photo-de-dimerization**. Dronpa protein can occur in two reversible stages: ON as a bright green tetramer (or a engineered dimer), or OFF as a non-fluorescent monomer under cyan light (405–450 nm), and back to ON stage after UV light excitation (390 nm). Light-induced dissociation used to control the activity of ITSN, a Rho GTPases regulator. The *caged* conformation—a Dronpa-ITSN-Dronpa targeted to the membrane via CaaX motif forming a tetramer. Cyan-light induces tetrameric dissociation unblocking ITSN active site and the formation of fillopodia. The process can be reversed by UV light.

Another photo-dissociating protein is Dronpa, a green florescence that changes conformation from tetrameric to monomeric, and back, depending on the wavelength of light excitation. Dronpa owes its name to “dron,” a ninja term for “*vanishing*,” and “pa” referring to photoactivation. The *dronpa* gene was discovered during a screening of cDNAs from stony coral *Pectiniidae sp*. (Ando et al., 2004). Dronpa protein may occur in two stages: ON as a bright green tetramer, or OFF as a dark monomer under cyan light (~500 nm) excitation. Furthermore, violet light (~400 nm) is able to restore tetrameric formation (Ando et al., 2004; Zhang and Cui, 2015). Note: a rationally designed dimeric Dronpa has been developed by site-directed mutagenesis (Zhou et al., 2012). These ON/OFF properties of Dronpa have been successfully used *in vivo* in zebrafish to visualize single neurons inside tissues. For this purpose, temporal neuronal-specific expression of Dronpa was achieved by mRNA transfer. Then, time-lapse imaging was performed by erasing the Dronpa fluorescence entirely, and re-highlighting it in a single neuron by violet-light. This procedure was repeated several times in order to reconstruct the entire neural network of zebrafish (Aramaki and Hatta, 2006). Besides Dronpa's light-switchable states, it has also been used to disaggregate proteins, mostly from a caged conformation—two Dronpa molecules flanking a functional domain. Dimeric or tetrameric Dronpa, upon cyan-light, dissociate, exposing the caged domain. For example, ITSN2 (Intersectin 2, Cdc42-specific mediated by guanine nucleotide exchange factor GEF) was fused to a Dronpa at each end. One of the Dronpa moieties had a membrane localization signal (CaaX). ITSN2, which normally activates Rho family GTPases—the master regulators of the actin cytoskeleton—, and is associated with centrosomes (Yeh et al., 2007; Rodriguez-Fraticelli et al., 2010), was inactivated by two flanking Dronpa caging. Upon cyan-light, Dronpa dissociated exposing the ITSN2 functional domain, prompting the formation of abundant filopodia within 30 min from illumination (Zhou et al., 2012; Figure 6). The same approach was used to generate a Dronpa-caged hepatitis C virus NS3-4A protease. Upon blue-light activation, protease activity was measured by the release of a mCherry-ss-CaaX from the cell membrane by cleaving its substrate site (ss) (Zhou et al., 2012).

### Parting for a new partner

UV-resistance locus 8 (UVR8) is an *Arabidopsis* photoreceptor protein. This protein forms homodimers that are photolabile and thus dissociate upon ultra-violet light (UVB) exposition (Chen D. et al., 2013). Photon absorption leads to conformational changes, which remain for several hours (Christie et al., 2012; Wu et al., 2012). After dissociation, monomers reversibly bind to partner COP1 (constitutively morphogenic 1) protein (Kim and Lin, 2013).

UVR8 has been mainly used to regulate transcriptional activation, where a DNA-binding domain fused to COP1 e.g., GAL4 DNA-binding domain-COP1, binds to a specific promoter, yet transcriptional activation is achieved by binding of photoconverted UVR8 fused to a transcriptional activator, such as NF-κB transcription domain—which causes a linear induction of a gene expression in mammalian cells (Crefcoeur et al., 2013).

The first light-triggered protein secretion method was developed using UVR8 protein properties. UVR8 was fused with *C*-terminal domain of well-documented secretory trafficking marker VSVG (vesicular stomatitis virus glycoprotein) and tagged with a fluorescent protein (FP). UVR8-VSVG-FP formed homodimers that were sequestered at the endoplasmic reticulum until photoactivation caused robust forward trafficking to the cellular membrane through the secretory pathway in neurons (Chen D. et al., 2013). This allowed the visualization of cellular markers and secreted cargo as it traverses the secretory pathway. Moreover, it circumvents the requirements of other tuneable secretion systems such as temperature changes or chemical-induction. An advantageous additional feature is that tryptophan-rich UVR8 domain requires no cofactor for photoreaction (Zhang and Cui, 2015), although exposure to UV light is a cause of concern, due to cellular and genetic damaging effects.

### Finding a partner when illuminated

The *HY4* gene of *Arabidopsis thaliana*, encoding a protein with characteristics of a blue-light photoreceptor, was first described by Margaret Ahmad and Anthony R. Cashmore in 1993 (Ahmad and Cashmore, 1993). CRY2 (blue-light receptor cryptochrome 2), product of *HY4*, is a member of photolyase-like blue-light receptors which mediates light responses in plants (Liu et al., 2008). CRY2 is the best characterized photosensor in *Arabidopsis*, where upon light activation physically interacts with, and activates, the transcription factor Cryptochrome-Interacting Basic helix–loop–helix 1 (CIB1) (Liu et al., 2011), an interaction that is reversed in absence of blue-light (Liu et al., 2013).

**Figure 7:**
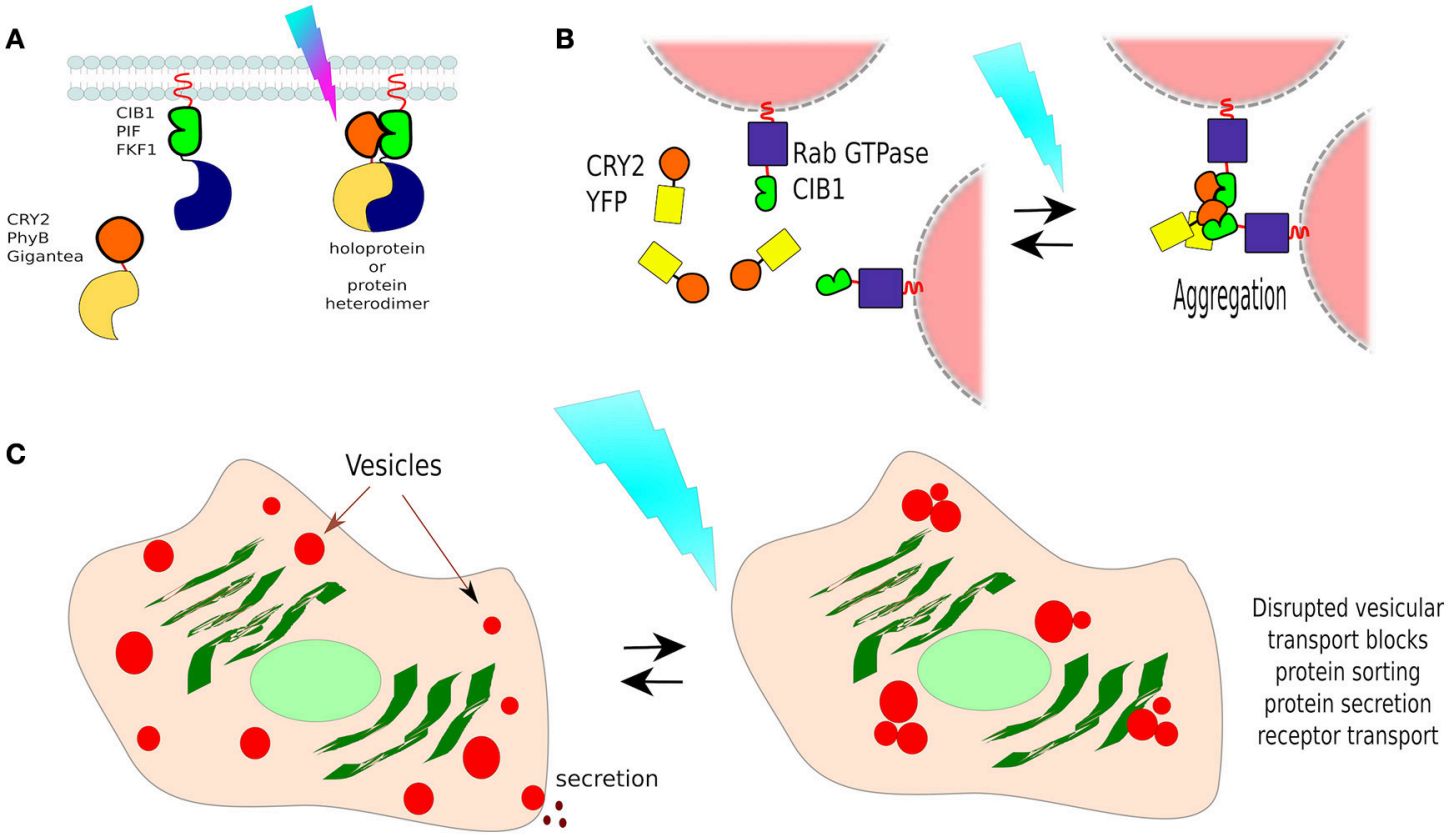
**Non-membranous opto-dimerisers. (A)** Dimerization by light. There are several available light-activated dimerisers such as CRY2 and CIB1 (others are portrayed too, see Figure 8). Heterodimerisation allows the coupling of complementary fragments or separated proteins upon photo-illumination. **(B)** Vesicle control by CRY2/CIB1. Different Rab GTPases, a family of proteins that bind to different membranes, were fused to CIB1 and co-expressed in cells with CRY2-YFP. In darkness, vesicle transport behaved normally, and CRY2 was found freely diffusing in the cell. Blue-light activation resulted in the aggregation of CRY2/CIB1-Rab GTPase as photoactivated-(pa)CRY2 not only binds to CIB1 but is also attracted to other paCRY2. **(C)** Control of vesicle-dependent processes. Rab GTPases mediate processes such as receptor transport, protein sorting, endocytosis, and protein secretion, thus using CIB1-Rab GTPase/CRY2-YFP it is possible to control such processes dependant on the chosen Rab GTPase.

The CRY2/CIB1 interaction was initially tested in mammalian cells by re-localizing proteins to the plasma membrane: CIB1 was fused with GFP and the CaaX prenylation motif for plasma membrane localization, whilst CRY2 was fused to mCherry. Blue-light resulted in the recruitment of CRY2-mCherry from the cytoplasm to the cell membrane of transfected HEK293 cells within seconds (Figure 7A). In the same work, the authors described the creation of a split CRE recombinase (see Box 3) that can form a functional enzyme when the two complementary halves are united by CRY2/CIB1 under blue-light in HEK293 cells (Kennedy et al., 2010).

In a different work, researchers showed that the CRY2/CIB1 could be used to photo-manipulate transport vesicles inside cells by fusing CIB1 to Rab GTPase, a protein that binds to vesicle membranes, and co-expressed with CRY2-YFP in cells. In darkness, vesicle transport behaved normally, and CRY2-YFP was found freely diffusing in the cytoplasm. Blue-light activation resulted in the aggregation of CRY2-CIB1-Rab GTPase, as photoexcited-CRY2 not only binds to CIB1 but also becomes attracted to each other (Figure 7B). Such *clumps* disrupt vesicular transport, suspending it until blue-light is turned off. In this way, the functions of specific Rab proteins in vesicular transport could be studied in neurons (Nguyen et al., 2016; Figure 7C). Rab GTPases mediate processes such as receptor transport, protein sorting, endocytosis, and protein secretion, thus using this method it is possible to control such processes, depending on the chosen Rab GTPase.

Since 2016, a second-generation CRY2/CIB1, having smaller proteins with reduced association in darkness and improved signaling states upon blue-light stimulation, is available (Taslimi et al., 2016). Yet, the conformation between CRY2 and CIB1 may prevent the interaction of cargo proteins. For example, Nihongaki and co-workers attempted to create a photoactivatable CRISPR/Cas9 using these partners carrying complementary halves of Cas9. However, that yielded no functional Cas9 and thus the authors chose a different pair of dimerising proteins, called *magnets* (see below).

Phytochromes B (PhyB) are chromoproteins naturally occurring in cyanobacteria and plants. PhyB binds to chromophore phycocyanobilin (PCB) as a cofactor that functions as a red-light sensor. Upon red-light exposure (~660 nm), PCB induces a conformational change in PhyB leading to its active form, PhyB_FR_. This form interacts with Phytochrome Interacting Factor (PIF), an interaction that can be reversed with even further far-red light (~740 nm; Ni et al., 1999; Kim and Lin, 2013). The Phy/PIF system has been used in mammalian cells to control the actin cytoskeleton where PIF was fused to a constitutively active ^*^Rac1 and this moiety was recruited to specific edges of the cell by photoactivating a PhyB tethered to the cell membrane. The result was the formation of protrusions (filopodia) from cell edges exposed to red-light (Levskaya, 2009), similar to the morphological changes to the LOV-Rac1 example described above.

Recently, the PhyB/PIF pair has been optimized for zebrafish by delivering a novel engineered PCB into embryos along with an optimized PhyB/PIF pair. Upon red-light exposure, membrane localized, PhyB-CaaX rapidly recruited PIF. Additionally, shifting the light to 750 nm released PIF back to the cytoplasm. To test whether the polarity of protein distribution could be manipulated at specific subcellular regions in living embryos, the authors generated a Pard3 (apical polarity protein 3)-PIF6 fusion protein, which could be directed by light to its binding partner, Pard6-PhyB-CaaX, at illuminated cell-to-cell joins, causing changes of polarity during neural tube development (Buckley et al., 2016).

One advantage of PhyB/PIF is its excitation wavelength, which is ideal for *in vivo* applications. Unfortunately, PhyB's chromophore, PCB, is absent in animal cells and thus should be exogenously supplemented which is a minor deterrent *in vitro* but a serious limitation *in vivo*.

The different wavelengths required for activation of optogenetic proteins makes feasible multi-chromatic inducible operations, as was achieved and reported by Müller et al. (2013) exploiting already existent optogenetic proteins. This was proved in single cells carrying three different gene reporter constructs and three different optogenetic dimerising proteins fused to transcriptional activators. These were: (1) a 311 nm UVR8/COP1 pair, where UVR8 was fused to macrolide-responsive repressor E, that binds near a minimal human cytomegalovirus promoter PhCMVmin, and COP1 fused to transcriptional activator V16 (Müller et al., 2013); (2) a Gal4-LOV-p65 (the activation domain of NF-κB transcription factor) which upon 465 nm light homodimerises and binds to the *CMV* promoter (Wang et al., 2012); and (3) a 660 nm PIF-TetR, a protein recognizing the TetO operator, and PhyB-V16 (Chen X. et al., 2013). Each of these constructs only induced specific gene expression (PhCMVmin-angiopoietin, *CMV*-vascular endothelial growth factor and *TetO*-firefly luciferase, respectively), in response to the correct wavelength, in a single cell (Müller et al., 2013).

**Figure 8:**
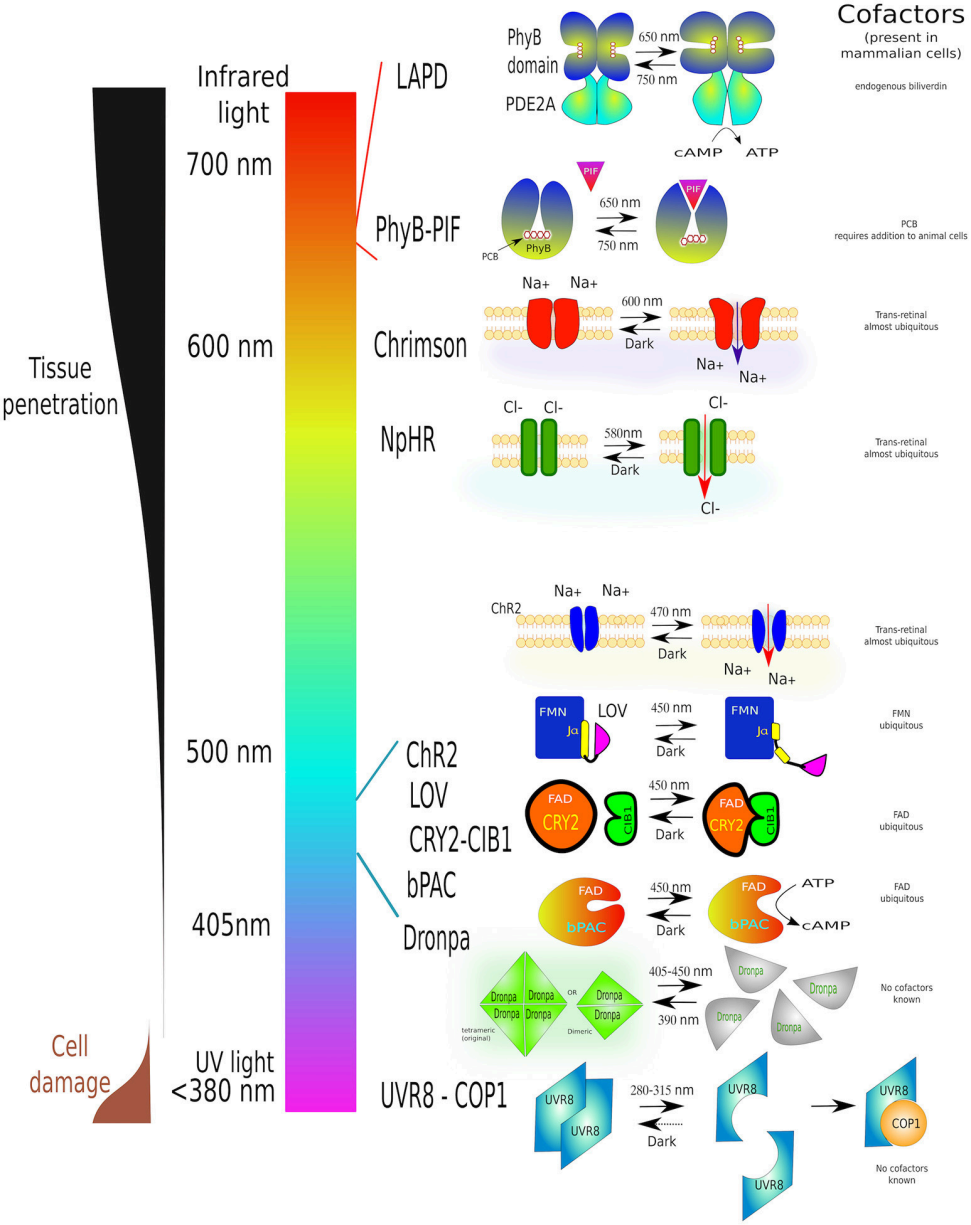
**A guide to some of the most commonly used optogenetic systems to-date**. Scheme of properties and conformational changes of several photoactivatable systems [phytochrome B (PhyB-PIF), red-light activatable phosphodiesterase (LAPD), Chrimson, Halorhodopsin (NpHR), Channelrhodopsin-2 (ChR2), LOV domain, adenylyl cyclase (bPAC), Dronpa and UV-resistance locus 8 (UVR8-COP1)]. Individual light-induced proteins have been assigned to their activation wavelength. Each system is shown before light stimulation (left) and after irradiation (right). Necessary cofactors are marked. Simultaneously this diagram shows the characteristics of different wavelengths on cell structures as well as tissue penetration. The detailed description of all these proteins can be found in the text this article. The image is partially based on (Zhang and Cui, 2015) and examples within the text.

Despite these impressive results, optogenetic techniques have limitations: they *leak* at different degrees in dark, heterodimerision might occur when the unstimulated proteins are overexpressed, and, in the case of protein dissociation, the two subunits must be at virtually identical levels, or the trapping one in excess, so there is no free active-subunit. Major challenges remain for the optimization of LOV-caged protein activity with no universal solution for them (Wu et al., 2009). Another disadvantage of the blue-to-orange excitation spectra is that they do not penetrate animal tissues as well as red-light (Jacques, 2013) which reduces their applications *in vivo*. Figure 8 summarizes several optogenetic actuators in relation to their activation wavelengths and characteristics.

The majority of somatic cells experience mechanical forces, namely pressure, flow, stretching, etc, during their existence. Cells respond to such mechanical forces in multiple ways but ultimately change their phenotypes and cellular activities. Therefore, mimicking physiological conditions is essential to understand cellular activities.

## Let the force be with you: regulation of mechanotransduction

A variety of methods, exist to analyse cellular responses to mechanotransduction. Noting that physical stimulation of cells is characterized by a low efficiency (Liu et al., 2016). Experimental strategies to induce mechanical stress include: fluid shear stress, where cells are exposed to changes in the perfusion and/or viscosity of fluids, and cell stretching—where the adhesion surface is stretched. Although, the advantage of these techniques is a very precise regulation of cell morphology, and the possibility of coupling these systems to high-resolution microscopy, the manipulation of specific mechanoreceptors at the molecular level is not possible.

To address this issue, functionalized magnetic nanoparticles (fMNPs) have been engineered to bind specific cellular mechanoreceptors, after which, using magnets, the fMNPs can be pulled into any given direction to trigger mechanoreceptor signaling (Etoc et al., 2013). In this manner, concrete mechanoreceptors can be specifically activated for downstream applications. It has been reported that fMNPs coated with Rho-GTPases, regulators of actin cytoskeleton, can be magnetically localized to one of the cell's edge, leading to local remodeling of the actin cytoskeleton and morphological changes in various cell lines (Etoc et al., 2013). Although this procedure is highly specific, it lacks spatial resolution in particular *in vivo*.

By combining nano- and photo-sensing technologies, the optomechanical actuator (OMA) was generated. OMA consists of gold nanorods coated with a thermoresponsive polymer which shrinks immediately upon near-infrared illumination, thereby applying a mechanical load to e.g., a membrane receptor attached to an immobilized ligand. Thus, allowing manipulation of receptor mechanics with high spatio-temporal resolution. This method exploits optomechanical actuation of transmembrane receptors that are involved in cell-cell and cell-matrix interactions. These optomechanically activated receptors triggered the local recruitment of the focal adhesion markers: paxillin, F-actin and vinculin which allow the precise control of focal adhesion formation, cell protrusions, cell migration and T cell activation, through the application of cyclic mechanical stimulation induced first by near-infrared illumination. OMA can also be used in combination with protein ligand receptors (Liu et al., 2016).

In all cases, mechano- or magnetic-stimulation involve specialized equipment and thus not commonly used (Liu et al., 2016). Moreover, these techniques are mainly focus on the engineering of chambers and/or functionalized nanomaterials, which are then used to study simple cultures or single cells. We refer to reader to a couple of specialized reviews on the subject (Humphrey et al., 2014; Iskratsch et al., 2014). fMNPs could be used *in vivo* under controlled conditions though.

A very elegant approach has been the adaptation of the Notch signaling concept. The Notch signaling pathway is an evolutionarily conserved cell communication mechanism present in most multicellular organisms. This pathway plays essential roles during cell fate determination in both during development and tissue homeostasis. The Notch pathway functions via mechanoactivation by any of Notch ligands (DLLs or JAGs) on the signaling (aka sender or sending) cell, which binds and activates the Notch receptor on the neighboring cell—the receiving (aka receiver) cell. This interaction results in conformational changes in Notch extracellular domain and the exposure of a cryptic region that is then proteolyticaly cleaved by disintegrin, ADAM metalloproteinase and finally, intracellularly, by gamma-secretase. The result is the release of Notch intracellular domain (NICD) which then translocates to the nucleus to function, together with a complex of other proteins, as a transcriptional activator (TA) (Kopan, 2002).

**Figure 9:**
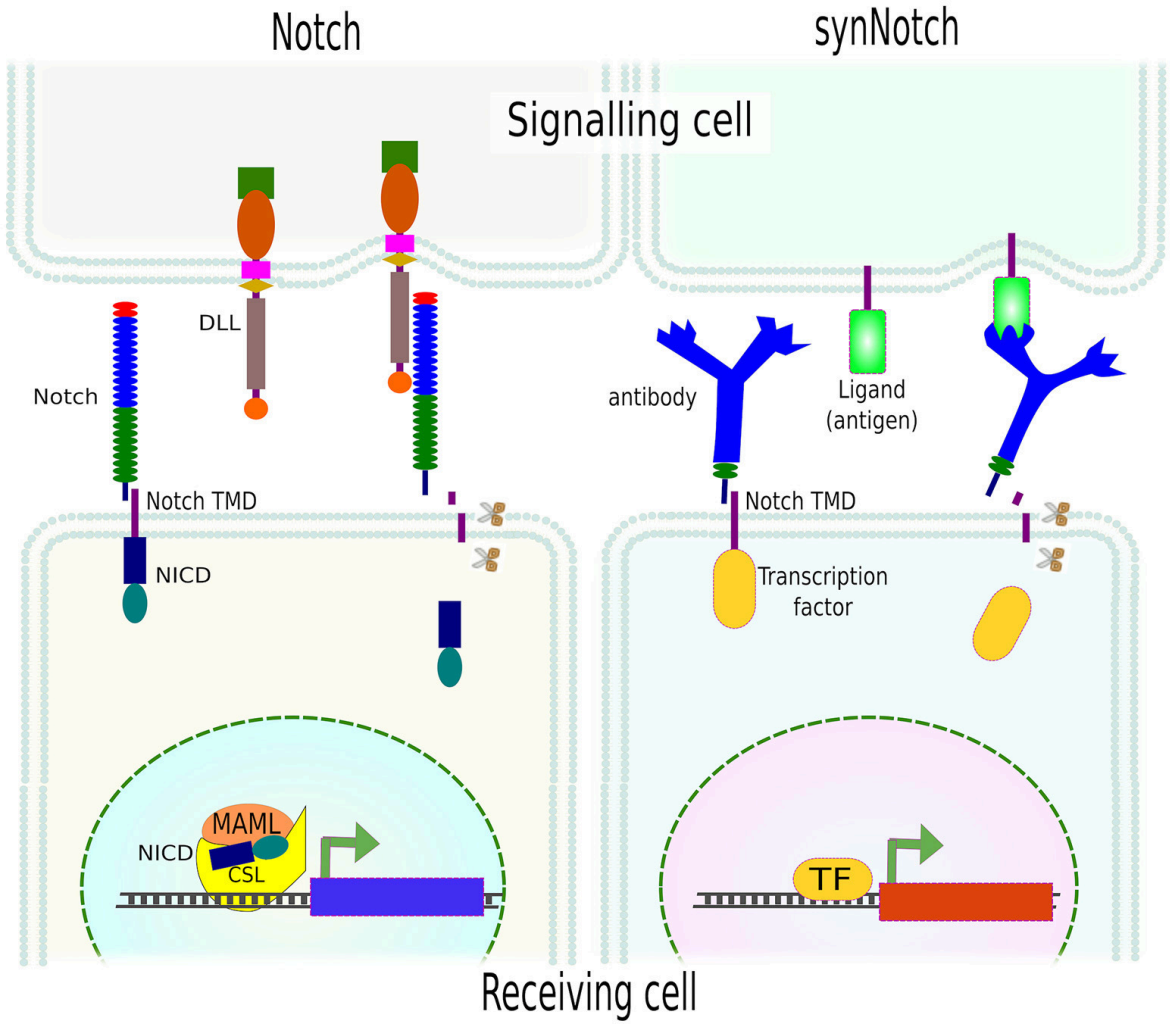
**SynNotch system**. Activation of Notch by its ligand (DLL) leads to mechanical forces and further proteolytical (scissors) cleavage of Notch extracellular domain (NICD) which then translocates to the nucleus and modulates gene expression. SynNotch exploits the mechanism of Notch receptor activation, using an antibody as an extracellular domain fused to the transmembrane domain (TMD) of Notch. Instead of NICD, different transcription factors can be used. The mechanical forces between the receptor (antibody) and the cell- or surface-attached ligand (antigen) resulted in the release of the intracellular domain (transcription factor) and the transcription of specific genes (reporters).

Instead of being limited to Notch receptors and Notch ligands, Morsut and collaborators engineered a series of constructs, called *synthetic Notch* (*synNotch*), where the extracellular domain of Notch was replaced by monoclonal antibodies, fused to the transmembrane domain (TMD) of Notch, followed by different transcription factors at the intracellular domain. As in Notch activation, the mechanical forces between the antibody and the surface-attached antigen resulted in the exposure of the cryptic region and the release of the intracellular domain (transcription factor). SynNotch was tested using a variety of transcription factors on cells that only upon presentation of the correct antigen, immobilized or presented by another cell, translocated to the nucleus and transactivated a reporter gene (Morsut et al., 2016; Figure 9). The flexibility of this system allows the generation of circuits and/or cell communication networks e.g., activation of one synNotch in a cell induced the expression of a membrane-bound specific antigen which in turn activated the neighboring cell(s) expressing the synNotch for this consecutive antigen. By this means, the authors created a multi-layered cell cascade using epithelial cells (Morsut et al., 2016).

Since the extracellular domain (antibody), ligand (antigen) and intracellular domain (transcription factor) are exchangeable, multi- and orthogonal-signaling are possible. The only limitation of this technique is the need for surface-attached ligand(s), in other words, an artificial environment needs to be generated which obviously limits *in vivo* applications. Yet, it can be used to unravel many other important biological questions, and pave the way for more complex synthetic circuits in mammalian cells which is needed for proper tissue engineering.

## Back to bases: genomic control

The modification of cellular behavior cannot be completed without the ability to modify the cell's genome and epigenome. As expected, the obvious design to modify the genome was by use of site-specific nucleases, although it was the advent of RNA-guided nucleases that has revolutionized the field completely.

Traditionally, gene control has been achieved by the use of vectors that carry genes under constitutive promoters. This view has gradually been replaced for tools that are able to erase, edit, or turn on or off endogenous genes. Genome editing, the availability to rewrite the information in the genome, is undergoing a craze due to a series of new and innovative methods that have made gene editing possible by virtually any lab.

The crucial breakthrough in genome editing was domestication of several naturally occurring DNA-binding proteins, and then their genetic modification, which lead to their sequence-specificity. Firstly, meganucleases, restriction endonucleases with long (14–40 bp) recognition sites, were meticulously mutated to recognize and cut desired sequences (Grizot et al., 2009). Yet, this is labor-intensive and the new restriction sites do not differ significantly from the original, further they might be promiscuous to their recognition sequences.

Instead of designing nucleases that recognize and cut DNA within the same domain, DNA-binding domains from different proteins could be used for sequence specificity, and then fused to a nonspecific nuclease. On this view, polydactyl zinc-fingers (ZFs) (Maeder et al., 2009; Gonzalez et al., 2010) were created. ZFs are domains found in transcription factors, DNA- and RNA-binding proteins that recognize a triplet of nucleotides. Thus, combining different ZFs, sequence-specific domains can be created. Due to their modular properties, and since they do not have nuclease activity on their own, ZFs were fused to the nuclease domain of the FokI restriction endonuclease and named ZF nucleases (ZFNs) (Kim et al., 1996). Locus specificity of ZFNs is determined by the ZFs, while. FokI, which needs to work as a dimer to cut DNA, functions as genetic scissors—two adjacent ZFNs, on opposite strands of DNA, are required to cause double strand brakes (DSB) (Urnov et al., 2010). Nevertheless, ZFNs have several limitations as there is no ZF modules for each of the 64 possible triplets.

In 2009, two independent groups reported the decoding of the Transcription Activator Like Effectors (TALEs) (Boch et al., 2009; Moscou and Bogdanove, 2009), natural type III effector proteins secreted by numerous species of the plat parasites *Xanthomonas spp*. These proteins modulate gene expression in host plants to facilitate bacterial colonization and survival. TALEs are beautiful modular proteins, each module identical to the others but in two residues (variable di-residues) (LTPEQVVAIASxxGGKQALETVQRLLPVLCQAHG) and each module binds to a single nucleotide in genomic DNA. In *Xanthomonas* there is an additional transcriptional activator (TA) attached to the *C*-termini of these proteins. Following ZFNs, TALEs have been fused to Fok1 to cut specific DNA sequences—called TALE nucleases or TALENs (Miller et al., 2011; Mussolino et al., 2011; Mussolino and Cathomen, 2012).

**Figure 10:**
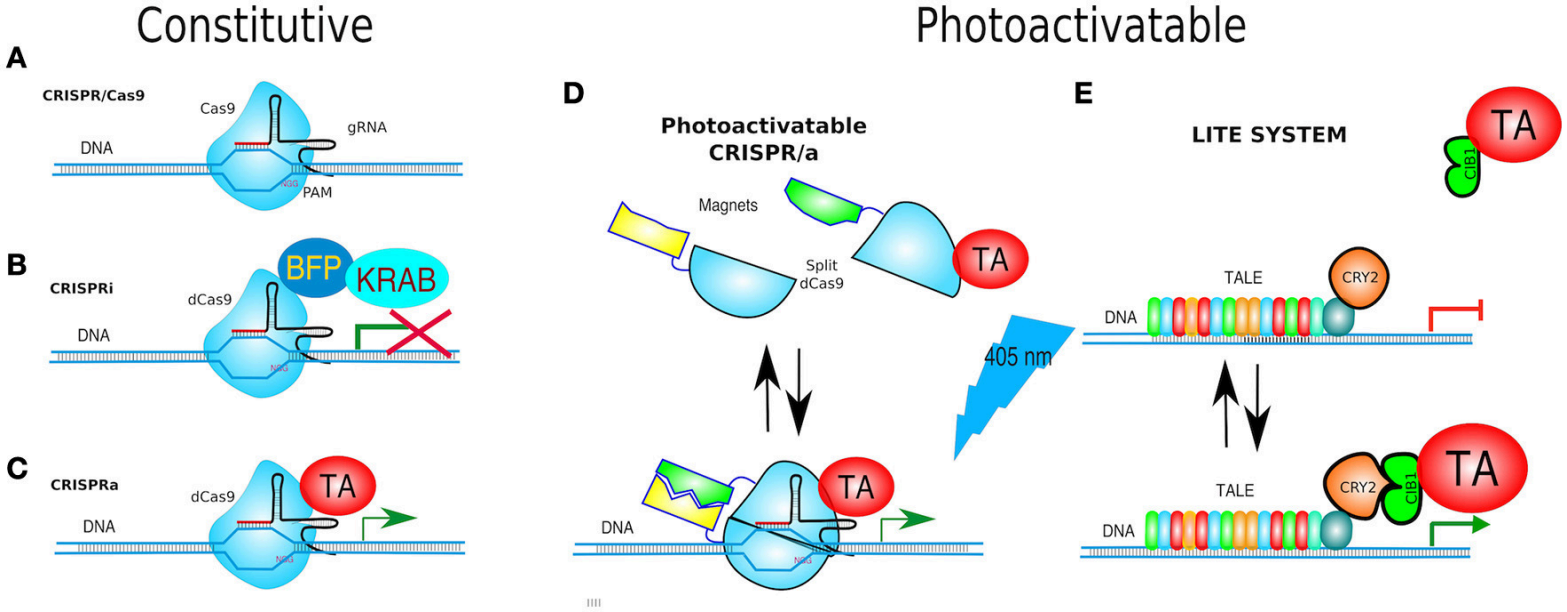
**Gene targeting. (A)** CRISPR/Cas9 is based on Cas9 endonuclease and its guide RNA (gRNA = crRNA and tracrRNA synthetic fusion). The 5′ end of the gRNA contains a sequence complementary to the target. Cas9/gRNA complex binds and cleaves specific DNA sequence only if followed by a PAM motive (NGG). **(B)** CRISPRi system utilizes fusion of dCas9, and DNA-binding domain (DBD), to a protein known to recruit repressive chromatin-modifying complexes, such as the Kruppel associated box protein (KRAB), which induces H3K9 methylation, resulting in virtually complete gene repression. **(C)** CRISPRa is used for gene transcriptional activation. dCas9 was fused to a transcriptional activator (TA) such as VP64 or VP128. **(D)** paCRISPR (photoactivatable CRISPR) utilizes photo-dimerising proteins called Magnets (positive—pMag and negative—nMag). Upon light activation, the split fragments of Cas9 [Cas9–N713 (residues 2-713) and C714 (residues 714-1,368)] are united to form a fully functional Cas9, i.e., a paCRISPR-TA for controlled transcriptional activation depicted. **(E)** LITE system enables modulating of gene expression by virtue of two components: TALE-CRY2, as a DBD, and CIB1 linked to either a TA e.g., VP64, or a transcriptional repressors (TR) e.g., KRAB. Upon blue-light TALE-CRY2, bound to DNA, dimerises to CIB1-TA or CIB1-TR inducing activation or silencing of gene expression, respectively.

TALEs have not only been used as nucleases but also, as in the original *Xanthomonas* scheme, fused with a transcriptional activator (TA) e.g., TBP (TATA-binding protein) (Anthony et al., 2014) or a transcriptional repressor (TR). Since TALEs bind specific regions of DNA without affecting it, they have also been coupled to optogenetic proteins for the activation or repression of gene expression, and named Light-Inducible Transcriptional Effectors (LITEs). LITEs modulate gene expression by virtue of two components: TALE-CRY2 which is designed to recognize a target locus (see below) and CIB1 linked to either a TA e.g., VP64, or a TR e.g., KRAB or p300^core^. TALE-CRY2 binds to DNA and upon blue light dimerises to CIB1-TA or CIB1-TR to induce activation or silencing of gene expression, respectively (Konermann et al., 2013; Figure 10E).

The advantage of TALEs and LITEs are their pliability, as many other *genomic editors* can be placed to study genetics and epigenetics (see below). Although, TALEs are genetically constructed by assembling almost identical modules, there are several excellent methods to facilitate their cloning, such as Golden Gate and LIC (Cermak et al., 2011; Schmid-Burgk et al., 2013). Addgene maintains an up-to-date list of techniques and TALE plasmid kits at https://www.addgene.org/talen/.

A better modular design could hardly be envisioned, but then however the type II CRISPR systems, which stands for Clustered Regularly Interspaced Short Palindromic Repeats, began to be understood (Mojica et al., 2009). Unlike previous designed-nucleases, type II CRISPRs combine RNA and protein—a nuclease—to target specific sequences of DNA, or RNA (see below).

CRISPR are the adaptive immune system of prokaryotes (bacteria and archae) and involve RNA-activatable nucleases. The activating RNA(s), sometimes one and sometimes two, guide the nuclease to the nucleic target, in most cases DNA. The components of CRISPR have been discovered in parts and has been enigmatic until recently. The first full description of CRISPR/Cas9 from *Streptococcus pyogenes* and *Streptococcus thermophiles* was achieved by two independent groups (Gasiunas et al., 2012; Jinek et al., 2012). They found that CRISPR/Cas9 required two RNAs to be able to target DNA. One of the RNAs, called *CRISPR-related RNA* (crRNA) encodes the target in its 5′ terminal, while the rest is partially complementary to a second RNA, the *transactivator of crRNA* (tracrRNA). The crRNA-tracrRNA complex is recognized by Cas9 and together they find the complementary match to the 5′ region of the crRNA.

The target, an invading phage's DNA, should have a complementary sequence to the crRNA 5′ end, followed by a protospacer adjacent motif (PAM), a short sequence that is not present in the crRNA (and thus absent in the prokaryote's genome) as a safeguarding element to avoid self-immunity.

CRISPR is, to date, considered the simplest and most efficient editors of (epi)genomes, since it involves no protein engineering (Sander and Joung, 2014; Barakate and Stephens, 2016). One of the most favorable features of these nucleoproteins complexes is that they unite **all the components of the** ***molecular biology central dogma*** (DNA-RNA-protein). This is virtually exclusive to them and thus many of the applications we will mention below are possible thanks to this fact.

Furthermore, CRISPR/Cas9 has been simplified by linking the two types of RNA into one single-chain **guide RNA** (gRNA, aka *single guide RNA* or sgRNA) (Jinek et al., 2012; Wang and Qi, 2016), and later on, adapted to gene editing in mammalian cells by Feng Zhang's and George Church's labs (Cong et al., 2013; Mali et al., 2013; Figure 10A).

Other simplifications involve (1) the co-expression of Cas9 and the gRNA from a single plasmid, the gRNA being expressed under a pol III promoter such as U3, U6 or H1, while Cas9 under the universal CMV promoter (Cong et al., 2013); (2) The expression of multiple gRNAs from a single transcript by use of either Csy4 endoribonuclease (Box 1) (Nissim et al., 2014; Tsai et al., 2014) or ribozymes—self-cleaving RNA domains (Box 2), or a highly conserved tRNA-processing mechanism—where endogenous RNases remove extraneous 5′ and 3′ sequences from the tRNA precursors (Carter and Wolfenden, 2015; Xie et al., 2015; Qi et al., 2016). Why multiple gRNAs? First because an increased number of targeting gRNAs enhanced mutagenesis efficiency in rice and maize (Xie et al., 2015; Qi et al., 2016), and second, because this allows multigene targeting (Li et al., 2013; Wang et al., 2013; Niu et al., 2014).

Box 1 **CSY4.**

Csy4 protein, first described in *Pseudomonas aeruginosa and Pectobacterium atrosepticum*, is an RNA endoribonuclease that processes CRISPR transcripts (pre-crRNAs) (Haurwitz et al., 2010; Przybilski et al., 2011). Csy4 recognizes a 28 bp spacer sequence (GUUCACUGCCGUAUAGGCAGcuaagaaa), where the last 8 nts can be variable (Tsai et al., 2014), on pre-crRNAs and cleaves immediately after the 20th nucleotide (Haurwitz et al., 2010, 2012; Sternberg et al., 2012). Due to the fact that Cys4 remains bound to the cleaved RNA, this enzyme can be used to cleave as well and binding RNA.

Csy4 has been used for producing gRNAs encoded in the 3'UTR mRNA of other genes expressed under the CMV promoter. One, or several gRNA is flanked by two Csy4 binding sites. Double cleavage by Csy4 releases the functional gRNA for further activation of CRISPR/Cas9 targeting of genomic loci. These tools were used for efficient modulation of endogenous promoters and implementation of tuneable synthetic circuits, including multi-stage cascades and RNA-dependent networks (Bikard and Marraffini, 2013). Csy4-based multiple gRNA generation for CRISPR applications has been applied in zebrafish, where several genes were simultaneously knocked out (Qin et al., 2015).

Box 2 **RNA selfies.**

Ribozymes are RNA molecules that can perform enzymatic reactions, usually self-cleaving. Due to this feature, they have been used to cleave mRNA in bacteria and eukaryotes. The finding that some ribozymes can be inhibited by small molecules like theophylline toyocamycin (Thompson et al., 2002; Yen et al., 2004; Kim et al., 2005) allows the control of such *riboswitches* (**Figure 12A**). Interestingly, photo-caged derivatives of toyocamycin exist, and they have been used for photochemical modulation of the protein expression in mammalian cells (Young et al., 2009).

Since ribozymes cleave RNA unaided, they have been used to excise gRNAs from the 3'UTR of reporter genes or even of *Cas9* to assure expression of both as well as to generate multiple gRNAs from a single transcript (see main text for details).

The use of nucleoside analogs (as toyocamycin) into cells causes significant side effects. Thus, the development of more specific strategies is necessary in order to use this approach in gene expression control.

Box 3 **Site-specific recombinases (SSRs).**

SSRs are sophisticated scientific tools that provide the possibility of precise manipulation of genomic DNA. Best known recombinases are: Vika, VCre, phiC31 integrase, Dre, Nigri/nox, Panto/pox, Flp, Dre and Cre (Sauer and McDermott, 2004; Karimova et al., 2016; Kawano et al., 2016). Once a recombinase recognizes two specific sites, it recombines them into one, excising the flanked DNA region. An exception of this rule is when the recognition sites are in opposite directions, in such case the area between them is inverted.

As recombinases require the specific insertion of recognition sites in the genome, and since they have been multiple times reviewed elsewhere, we will just mention that these methods have also been modified so they can be regulated: (1) by chemicals, examples are the tamoxifen-inducible Cre–ER2 by binding a mutant estrogen receptor to prompt translocation of Cre into the nucleus, or rapamycin-CID of split Cre (Banaszynski et al., 2005). (2) by optogenetics by use of complementing Cre fragments fused to either magnets or CRY2-CIB1 (Feil et al., 1997; Jullien, 2003; Kennedy et al., 2010; Duyne, 2014; Gonzalez et al., 2015; Kawano et al., 2016).

As pol III promoters cannot provide tissue-specificity expression of gRNAs, producing multiple gRNAs has been made possible by incorporating gRNAs into the 3′UTR of Cas9 mRNA—regulated by a pol II promoter, and flanking them with ribozymes (Yoshioka et al., 2015; Xu L. et al., 2016), or Csy4 recognition sites (Box 1; Tsai et al., 2014).

CRISPR/Cas9 has already demonstrated its effectiveness to modify endogenous genes of various bacterial, plant or animal organisms, and currently there is the first human clinical trial (https://clinicaltrials.gov/ct2/show/NCT02793856?term=crispr&rank=4). In addition, it should be noted that gRNA libraries have been successfully implemented in studies of the function of coding (Chen et al., 2015) and non-coding RNAs (Copeland et al., 2001; Kim and Kim, 2014; Ma et al., 2014).

Targeting of specific loci allows the disruption of genes by indels (short *in*sertions or *del*etions) during double-strand break (DSB) repair, as well as the creation of seamless knock-ins, point mutations, and more. Nonetheless, gene correction and knock-ins are still too inefficient for most clinical applications.

### A CRISPR regulator

Nuclease-dead Cas9 (dCas9) is catalytically inactive—due to two point mutations in its nuclease domains—each cutting a single DNA strand. Yet, it retains its gRNA binding and DNA target capabilities. Therefore, it becomes an RNA-guided DNA-binding protein (Gilbert et al., 2013; Larson et al., 2013). Similarly to the abovementioned TALEs fused to transcriptional effectors, dCas9 has been adapted for transcription modulation of endogenous genes by either turning them OFF (CRISPRi, CRISPR interference) or ON (CRISPRa, CRISPR activator) and even fused to Fok1 to generate duoble-dCas9-Fok1 that were expected to have less off-target effects (Guilinger et al., 2014; Tsai et al., 2014).

Turning genes off has been achieved solely by targeting dCas9 to the promoter of a gene, although this often results in only partial disruption of gene transcription (Mali Prashant, 2014). CRISPRi goes one step further by fusing dCas9 to a protein known to recruit transcriptional repressors (TRs) such as chromatin-modifying chromo shadow domain of HP1α, the Kruppel associated box protein (KRAB), or the WRPW domain of Hes1, resulting in virtually complete gene repression (Qi et al., 2013; Keung and Khalil, 2016; Figure 10B). Additionally, for better results, the scaffold of gRNAs have been modified by inclusion of RNA stem-loops that are specifically bound by RNA-loop-binding proteins (see Table 1), such as MS2, fused with TRs (Zalatan et al., 2015; see Table 1 and **Figure 12B**). CRISPRi can silence both coding and noncoding genes, such as long noncoding RNAs (lncRNAs) and microRNA (miRNA) (Gilbert et al., 2013, 2014; Larson et al., 2013; Zhao et al., 2014). Moreover, CRISPRi can be applied to organisms that lack the RNAi machinery such as *Saccharomyces cerevisiae* (Drinnenberg et al., 2011).

**Table 1:** **RNA loops and binding proteins**.

**RNA loop**	**RNA-loop binding protein**	**References**
MS2-binding site (MBS)	MS2 coat protein (MCP)	Bertrand et al., 1998; Romaniuk et al., 1987
Lambda boxB RNA sequence	Phage lambda N protein (λ_N22_)	Daigle and Ellenberg, 2007
QB Stem-loop	QB coating protein	Rumnieks and Tars, 2014
PP7 RNA sequence	PP7 coating protein	Lim et al., 2001; Lim and Peabody, 2002
Box B nut L/R	phage HK022 Nun protein	Chattopadhyay et al., 1995; Van Gilst et al., 1997
Amino-terminal RNA-binding domain of U1 snRNP A (U1A)	U1A protein	Moras and Poterszman, 1995; Oubridge et al., 1994
Nanos Response Elements (NRE)	NRE-specific protein e.g., Pumilio	Murata and Wharton, 1995; Wharton and Struhl, 1991

*RNA 3D structure is highly complex, which is associated with a number of its functions. RNA loops are structures which result from base pairing and can perform multiple functions such as binding to proteins, RNA or DNA. RNA-loops have been used to tag mRNA (Figure 12B) as well as to bring transcriptional regulators to the modified gRNA-loops of CRISPR/Cas9 (see text for more details). Here, we list the stem-loop structures known to bind to specific proteins*.

At the other end, CRISPRa is used for gene transcription activation (Gilbert et al., 2014), where dCas9 has been fused to a TA such as VP64 or VP128 (Larson et al., 2013; Figure 10C). A *potentiated* CRISPRa exists, where dCas9 was modified with a tandem peptide tail that is then recognized by an intracellular antibody fused with a TA (VP64) (called SunTag) (Farzadfard et al., 2013; Tanenbaum et al., 2014). The multimerisation of TAs has also been achieved by use of dRNAs (dead gRNAs). dRNAs contain 14- to 15-bp target sequences and MS2-binding loops and can activate gene expression without inducing DSBs even when using an active Cas9. Originally, it was used for orthogonal gene knockout and transcriptional activation in human cells (Dahlman et al., 2015).

Since the CRISPR/Cas9 and dCas9 systems are always active, modifications to make them inducible are desirable. Once again, the techniques discussed above came to aid.

The first **inducible CRISPR** was a split-Cas9 composed by the *C*-terminal fragment and *N*-terminal fragment of Cas9—Cas9(C) and Cas9(N)—fused to FKBP or FRB, respectively. In the presence of rapamycin, Cas9(N)-FRB dimerises to Cas9(C)-FKBP formed a functional Cas9 (Banaszynski et al., 2005; Zetsche et al., 2015b). Since spatial separation of the fragments can decrease background activity, caused by spontaneous auto-assembly of Cas9, Cas9(N)-FRB was directed to the nucleus via two nuclear localization signals whilst Cas9(C)-FKBP carried a nuclear export signal.

Using the tet-inducible expression system, named Tet-on/Tet-off, a doxycycline-inducible Cas9 was generated (González et al., 2014; Dow et al., 2015). Upon addition of doxycycline, Cas9 was expressed to introduce monoallelic and biallelic indels, as well as frame shift mutations, in multiple target loci (Dow et al., 2015). However, this method is slow as it requires transcription and translation of Cas9.

As in other chemical-induced methods, the use of small molecules produces adverse effects such as those of rapamycin/doxycycline mentioned above. Another disadvantage arises from slow diffusion, causing difficulties in their rapid removal (Nihongaki et al., 2015). Finally, in addition to the side effects, the use of chemicals result in universal targeting and dose-responses that cannot be properly controlled *in vivo*.

Since no other method outclasses the spatiotemporal precision of optical stimulation, a photoactivatable Cas9 (paCas9) was designed based upon the split design (above) but instead using the CRY2/CIB1 optogenetic pair. Which was unsuccessful at first, but the group of investigators lead by Nihongaki replaced CRY2/CIB1 with other photodimerising proteins called Magnets (positive—pMag and negative—nMag)—previously reported by Kawano et al. (2015). Upon light activation, the split fragments of Cas9, (Cas9N) (residues 2-713) and Cas9C (residues 714-1,368), were united to form a fully functional Cas9. Both possible conformations—Cas9N-pMag/Cas9C-nMag and Cas9N-nMag/Cas9C-pMag showed light-triggered Cas9 activity. All other features of full-length Cas9 remained unaffected, such as the PAM specificity and its nuclease activity on genomic DNA. The paCRISPR was also adapted for transcriptional activation (paCRISPRa) (Nihongaki et al., 2015; Figure 10D).

Due to the rapid Magnets' dissociation properties (Nihongaki et al., 2015), paCRISPR is likely not fully amenable to *in vivo* applications, which would require long photostimulation periods and thereby the immobilization of the organism. In this vein, a system where light would induce a long-lasting effect would be far more desirable.

### More to CRISPR

The resounding success of CRISPR/Cas9 has triggered a search for other CRISPR techniques that could work better or distinctively. This has led to the discovery of a shorter Cas9 that potentially can fit in viral genomes other than lentiviruses for gene delivery and therapy (Friedland et al., 2015). Another discovery was **Cpf1** which is a type V CRISPR effector endonuclease.

The most notable features of CRISPR/Cpf1 are: the absence of tracrRNA, its crRNA (42–44 nucleotides) shorter than Cas9 gRNA (more than 100 nucleotides), reducing costs (Fagerlund et al., 2015), and there are significant differences in the PAM sequences of Cpf1 and Cas9 i.e., 5′-T-rich vs. 3′-G-rich, respectively. Furthermore, Cpf1 uses an entirely different mechanism of target recognition, and cleaves producing DNA over-hangs (Zetsche et al., 2015a; Gao et al., 2016), unlike Cas9 which makes blunt cuts. Thus, Cpf1 has been suggested as an alternative to the classic CRISPR/Cas9. It is early days and there still is some controversy on the efficacy of Cpf1, having groups reporting great effectiveness (Kleinstiver et al., 2016) whereas others do not (Kim et al., 2016; Tóth et al., 2016). Undoubtedly, the specificity of Cpf1 is one of its greatest opportunities as an additional gene editor, in particular for AT-rich regions, as well as for gene editing of protozoa or organisms that have genomes with high AT content.

These CRISPR tools may be used in combination e.g., in studies on gene regulation, when there is need to target different sequences, which can be achieved using different gRNAs and crRNAs (Fagerlund et al., 2015).

Editing genes is a sound approach to study their functions, yet cell control requires that gene expression is modulated, which can only be accomplished via epigenetic editing.

## Nature or nurture?

Genomic accessibility is controlled by a series of epigenetic modifications that include DNA methylation, and histone methylation or acetylation. DNA is methylated by methyl-transferases (DNMT) and demethylated by TET hydroxylases, while histones are acetylated by histone acetyl-transferases (HATs), deacetylated by histone deacetylases (HDACs), methylated by histone methyl-transferases (HMTs) and demethylated by histone demethylases (HDMs) (Arrowsmith et al., 2012; Kooistra and Helin, 2012; Jin et al., 2015).

**Figure 11:**
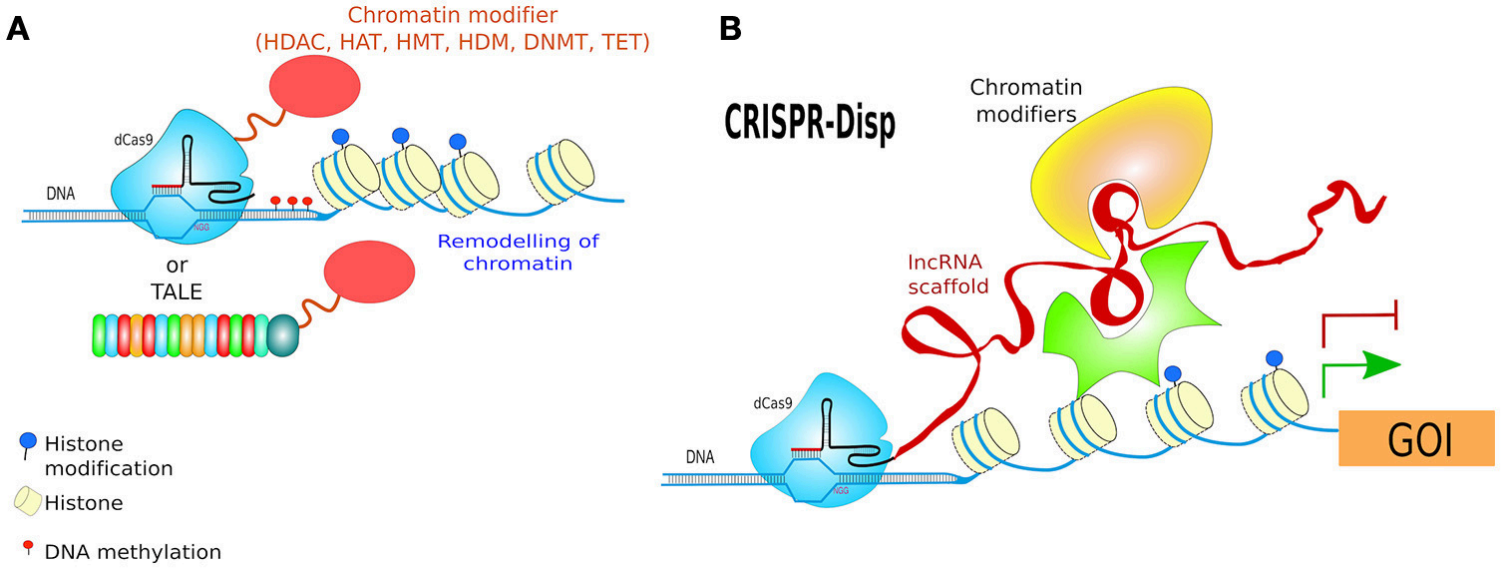
**Epigenetic modulation. (A)** CRISPR/TALE-targeted chromatic modification, dCas9 or TALEs as DNA binding domains may deliver several modifiers (epigenetic editors) which can modify DNA or chromatin structure and thus regulate expression of endogenous genes. Using these methods several epigenetic modifications have been achieved such as DNA methylation or demethylation, histone acetylation or methylation depending of the epigenetic editor used. **(B)** CRISPR-Display (CRISPR-Disp) uses the properties of long non-coding RNA molecules, which can act as scaffold for effectors (see Figure 12A). The functional RNA domains fused to gRNAs at multiple points, what allowed the display of RNA-protein complexes to genomic loci.

DNA methylation of CpG islands is often found in the promoters of non-expressing genes, and thus hypothesized as a repressing mark. To determine if this assumption was correct, two independent research groups used TALEs or dCas9 to deliver the catalytic domain of Ten 11 Translocation hydroxylase (TET1) to methylated CpG regions of promoters in mammalian cells. Demethylation of CpG islands lead to upregulated gene expression of endogenous genes, proving that DNA methylation indeed functions, in most cases, as a repression mark (Maeder et al., 2013; Xu X. et al., 2016; Figure 11A).

Histone-3 lysine 9 (H3K9) di- or tri-methylation has been correlated to the compaction of chromatin and gene repression. During cell differentiation, for example during EMT, epithelial cells gain of mesenchymal cell characteristics by repression of epithelial-specific genes such as *E-cadherin*. Repression of this gene has been attributed to H3K9 di-methylation by Snail1. In this vein, Cho and collaborators tested the effects of methylation of histones by using TALE-TSET (a chimera of the E-Box region of Snail binding site with the SET domain of EHMT – an HMT). The increase of histone H3K9 di-methylation repressed the expression of the target gene, *E-cadherin*, triggering a more migratory and invasive cell phenotype as expected (Cho et al., 2015).

Acetylation of histone 3 lysine 27 (H3K27), a mark often associated with open chromatin, has also been achieved using a fusion of dCas9 with human acetyltransferase p300 targeting the promoters of the following genes: *MYOD (*Myogenic Differentiation 1), *IL1R* (Interleukin 1 Receptor Type 1), *OCT4* (Octamer-Binding Protein 4), which provoked transcriptional activation of the downstream genes (Hilton et al., 2015).

Several other epigenetic modifications have also been rewritten, such as reduced histone acetylation by dCas9-LSD1—an HDM; histone methylation (H3K9 tri-methylation) by dCas9-KRAB (CRISPRi) (Figure 10B); or CpG methylation by dCas9-DNMT3A—DNA methyltransferase (Kearns et al., 2015; Thakore et al., 2015; Vojta et al., 2016; Figure 11A). For a recent review documenting more examples refer to (Laufer et al., 2015).

Since epigenetic marks play an essential role in cell differentiation, these techniques have enormous potential for *in vivo* applications. Modifications such as LITE or paCRISPR will surely be applied to control specific cells at specific times e.g., during embryo development for a more detailed understanding of epigenetic regulation and function.

We consider RNA as the last frontier when it comes to manipulation, this is largely due to the plethora and complexity of RNA types and functions, and the continuously changing conformation of these molecules. Most research has focused on degrading mRNA but new and more powerful techniques are appearing that could also revolutionize how we can manipulate RNA.

## Controlling RNA

Using small oligonucleotides, such as endogenous (miRNA) (Fabian et al., 2010), synthetic RNA (shRNA, siRNA), or other nucleic acid analogs (locked nucleic acids LNA, morpholino, 2′-O-methyl RNA oligo) (Cooper et al., 2009), it is possible to block gene expression. Despite their popularity in cell biology, they can hardly be regulated, and their effects often account for partial mRNA-targeting destruction—something that gene editing tools can outperform at the genomic level resulting in no gene at all (Evers et al., 2016). Additionally, new gene editing tools that target RNAs have been found.

Whilst the most of known prokaryotic adaptive immune systems target DNA substrates (Brouns et al., 2008; Wright et al., 2016), type III and VI CRISPR systems, e.g. **C2c2**, directly interfere with single-stranded RNA (ssRNA) targets carrying complementary protospacers (Shmakov et al., 2015; Abudayyeh et al., 2016). Thus, C2c2 can be programmed to cleave specific RNAs.

RNA cleavage by C2c2 is mediated by catalytic residues found in the two conserved HEPN domains (Higher Eukaryotes and Prokaryotes Nucleotide-binding). Double and quadruple HEPN mutations (R472A, H477A, R1048A, and H1053A) (dead-C2c2, dC2c2) did not affect C2c2 pre-crRNA cleaving activity, whilst all these mutants could still bind to target RNA (Abudayyeh et al., 2016). The ability of C2c2 to produce its own gRNAs allows co-expression of *C2c2* and multiple-gRNAs from RNA polymerase II promoters for tissue-specific expression *in vivo* (East-Seletsky et al., 2016). Up to now, there is no report to the best our knowledge of any adaptation of C2c2 to eukaryotic cells.

## Decoding the non-coding

**Figure 12:**
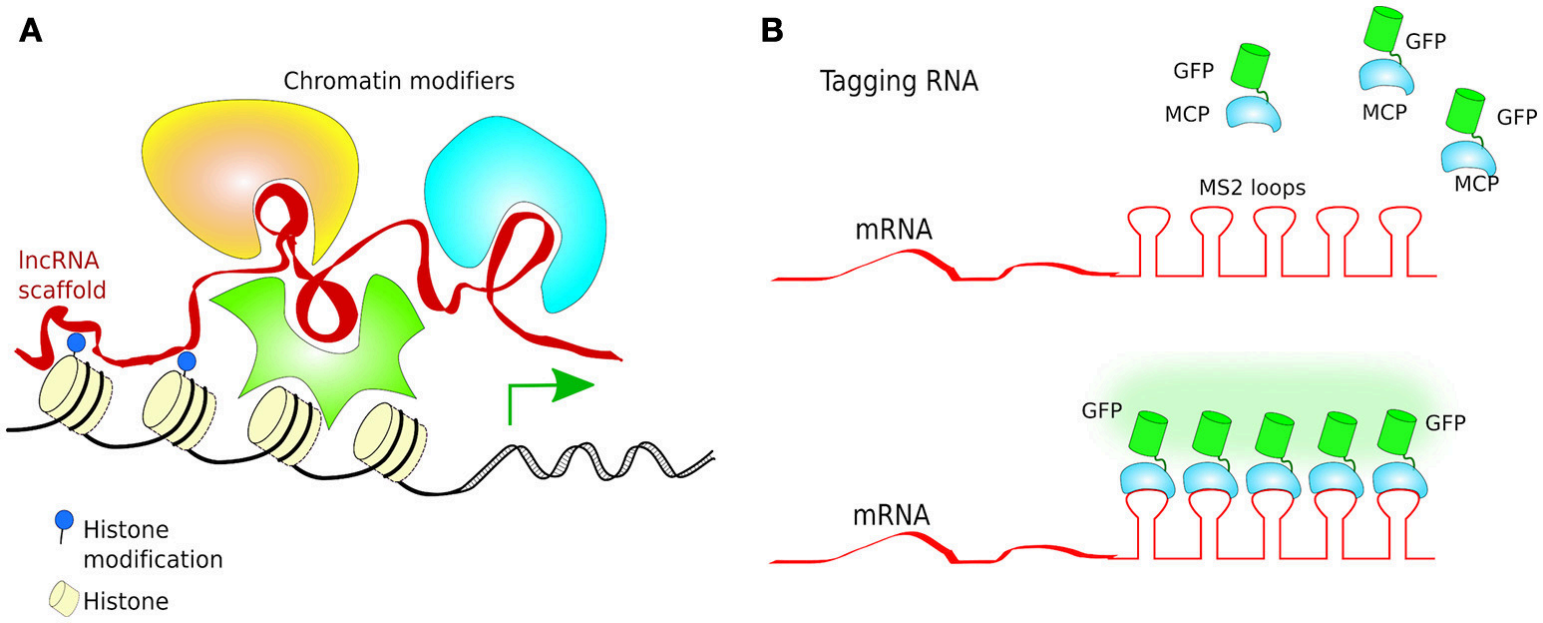
**RNA localization and function. (A)** Long non-coding RNAs (lncRNAs) play essential roles on the regulation of chromatin structure. Some lncRNAs may act as scaffolds for several effector proteins which can modify chromatin organization via recruiting chromatin modifiers; **(B)** Tagging RNA for tracking and visualization in living cells is possible by using RNA loops and fusion protein fusion which contain RNA loop-binding proteins fused to fluorescent proteins (FPs) such as GFP.

Among the non-coding RNAs (ncRNAs), a group that includes siRNAs, miRNAs, circularRNAs, and piRNAs, there is a heterogeneous group called long non-coding RNAs (lncRNAs)—RNA molecules longer than 200 nucleotides that do not code for proteins. LncRNAs are, in fact, the largest ncRNA transcript class in mammalian cells, with approximately 10,000 lncRNA genes so far annotated in humans. LncRNAs are emerging as crucial regulatory factors in cells, as they interact with DNA, proteins and other RNAs (Espinosa et al., 2016). Although the roles of most lncRNAs are far from being elucidated, the number of described lncRNAs is increasing and many reports suggest they participate in positively or negatively regulation of gene expression during development and differentiation as well as in disease conditions (Kornienko et al., 2013; Zhao and Lin, 2015). Some lncRNAs function as scaffolds that assemble protein complexes to activate or inactivate certain cellular functions (Lee et al., 2016; Figure 12A). Others function as guides for protein complexes to genomic loci, while a few others are known to serve as decoys that remove proteins from target genes (Yang et al., 2015). A note to the reader: there are potentially many annotated lncRNAs that do code for proteins or peptides (Espinosa et al., 2016; Nelson et al., 2016), although the occurrence and relevance is currently unknown.

To investigate these capabilities, researchers fused functional lncRNA domains (up to 4.8 kb) at different positions (5′, middle or 3′) of gRNAs so they could guide dCas9 to genomic loci of interest. Testing different architectures of CRISPR/dCas9 complexes to *display* fragments of lncRNAs (CRISPR-Disp) such as protein-binding cassettes, aptamers or pools of random sequences, it was shown that the scaffolding abilities of lncRNAs could be manipulated and targeted to genomic regions of interest for the control of e.g., gene expression (Figure 11B). According to the authors, CRISPR-Disp could be potentially used to ectopically target functional RNAs and ribonucleoprotein complexes to genomic loci (Shechner et al., 2015). Currently, little else has been undertaken to deal with lncRNA to control cellular phenotypes. However, the combination of lncRNAs to inducible systems (based on optogenetics or small compounds) would be required for a better understanding of the roles of lncRNAs in biology and their further applications. LncRNAs are difficult to study, due to their multiple domains which are able to bind to other RNAs, proteins, DNA and even small compounds, yet for the very same reason, being able to control them is essential to further understand cellular functions.

The use of RNAs as scaffolds or guides for proteins and/or RNAs, would require a more detailed understanding of the changing structure of RNAs. Yet, examples of RNA domains that are recognized by proteins are known, such as the RNA stem-loops (structures occur in single-stranded RNA molecules resulting from unpaired nucleotides in the middle of complementary sequences) that are recognized by a series of proteins (Table 1). These loops/recognizing proteins have been used to tag RNA for localization and tracking (Figure 12B), as well as to target mRNA localizations to specific cell compartments e.g., b-actin mRNA with multiple MS2 stem–loops in its 3′ UTR was dragged to focal adhesion sites by a fusion of MCP (MS2 loop binding partner) to the focal adhesion plaques protein vinculin, resulting in high local translation of b-actin and larger adhesion plaques (Katz et al., 2012).

In fact, long synthetic RNA scaffolds have already been used for the modulation of the metabolism of bacteria. Engineered RNA blocks which contain the PP7 and MS2 binding domains (Table 1), were assembled into multidimensional scaffolds with distinct protein-docking sites. These self-assembling RNA scaffolds enabled the organization of the oxidoreductive enzymes, resulting in an increase in hydrogen output in bacteria—mainly by increase of the rate of electron transfer between enzymes (Delebecque et al., 2011). A similar strategy was used in *E. coli* to increase the metabolic output of pentadecane production by immobilization of enzymes involved in fatty aldehyde and succinate production into a synthetic RNA scaffold. The authors suggested that intra-cellular scaffolding of multi-enzymatic reactions could enhance the direct passing of intermediary metabolites from one enzyme to another, to increase the amount of the final product of the pathway (Sachdeva et al., 2014). To the best of our knowledge, synthetic RNA scaffolds have not been shown to function in mammalian cells.

## Concluding remarks

The discovery of a multitude of different proteins, RNAs, and systems, from all over the kingdoms of life, have brought a wealth of new “domains” that can be adapted for the generation of novel tools in molecular and cell biology.

There is need for improvements in these techniques to the full implementation in multicellular organisms. Protein dimerization (CID) and destabilization (DD) have been used to some degree in animals showing their potential but with many shortcomings too. Several of CID/DD obstructions have been overcome by optogenetic systems, some of which, e.g., ChR2/NpHR, have already been used to control specific behaviors in rodents with astonishing results (Root et al., 2014). This is due to the very natural functioning of neurons and the light activating responses of these receptors. Optogenetics, a field just in its infancy, is already running wild and making strides in our understanding of living organisms with an incredible precision. This field will surely provide many more surprises and beautiful tools to study living organisms at the molecular level in a detail that has never been previously achieved. Comprehensive cell control will be required for tissue and organ bioengineering, as foreseen by the initial prototypes, such as the photocontrollable ray fish cyborg (Park et al., 2016).

We expect the discovery or creation - by a collective effort between *in silico* modeling, bioinformatics and molecular biology—of far-red responding proteins, for better penetration in tissues, proteins which respond to uncommon wavelengths—plenty of room in the green/yellow and the far-red regions (see Figure 8), for orthogonal molecular activation in the same cell, as well as smaller and more dynamic domains which could be easily delivered via viral particles.

The convergence of several of the above-mentioned techniques will likely be used to create synthetic circuits resembling natural networks but externally manipulated with minimal invasiveness, a field rapidly evolving in prokaryotes, but yet to make an entrance into eukaryotes. This will require modification of endogenous loci, insertion of multiple gene moieties, tightly regulated promoters, feedback mechanisms, minimal/no unwanted effects, and lack of leakage under non-stimulating conditions. Finally, activation should involve minimal disturbance or stress, with no side effects.

What occurred to us when planning this review was to provide the reader with a *Swissknife* of cutting-edge molecular biology techniques to trigger the readers' imagination to apply them to their own research, and, in some cases, to make improvements to this toolkit.

The aging process seems to be accompanied by progressive changes in epigenetic information (Pal and Tyler, 2016). Thus, besides cell differentiation, control over the reversible nature of epigenetic modifications may well lead to a better understanding of the molecular mechanisms of aging, and potentially help find targets for drug development or biomedical interventions.

The control of cellular processes should also be applied to other organisms, in particular organisms that can create structures such as diatoms. Controlling diatoms' frustules shape could generate biocompatible materials that self-assemble into macro structures for biomedical or industrial purposes.

The potential therapeutic uses of these techniques, one might envision gene repair, gene removal e.g., HIV, therapeutic viral self-deleting genome, genome restructuring or correction of chromosomal polyploidy. We might go further to suggest that studies on the origin of life could be supplemented with, literally, new light, by using some of these novel systems.

All in all, the future of cell control is *optogenetically* bright; there are many challenges ahead, mostly in areas such as RNA-control and the role of epigenetic mechanisms to fine-tune chromatin structure, and cellular function. Gene repair is still too inefficient at this point, therefore, modifications to improve the performance of gene editing tools in accuracy and efficiency are eagerly awaited, in particular.

The systems described in this review are a good start to envision the future directions in cell biology and bioengineering. The final goal is to be able to answer biological questions that have eluded us for centuries and to ultimately generate controllable living entities not only for biosafety and biopharmaceuticals but for biomaterials and tissue engineering.

## Author contributions

JC, MKi, JK, MKo, and AR collected the information, organized the text and wrote the manuscript. JC, MKi, JK, AS, and AR conceptualized the structure and content of the manuscript. AR prepared all figures. AS and AR coordinated the work. All authors read and approved the final manuscript.

### Conflict of interest statement

The authors declare that the research was conducted in the absence of any commercial or financial relationships that could be construed as a potential conflict of interest.

## References

[B1] Abudayyeh O. O. Gootenberg J. S. Konermann S. Joung J. Slaymaker I. M. Cox D. B. T.. (2016). C2c2 is a single-component programmable RNA-guided RNA-targeting CRISPR effector. Science 353:aaf5573. 10.1126/science.aaf5573 27256883 PMC5127784

[B2] Ahmad M. Cashmore A. R. (1993). *HY4* gene of *A. thaliana* encodes a protein with chracterostics of a blue-light photoreceptor. Nature 366, 162–166. 10.1038/366162a0 8232555

[B3] Airan R. D. Thompson K. R. Fenno L. E. Bernstein H. Deisseroth K. (2009). Temporally precise *in vivo* control of intracellular signalling. Nature 458, 1025–1029. 10.1038/nature07926 19295515

[B4] An W. Jackson R. E. Hunter P. Gögel S. Van Diepen M. Liu K.. (2015). Engineering FKBP-based destabilizing domains to build sophisticated protein regulation systems. PLoS ONE 10:145783. 10.1371/journal.pone.0145783 26717575 PMC4696822

[B5] Ando R. Mizuno H. Miyawaki A. (2004). Regulated fast nucleocytoplasmic shuttling observed by reversible protein highlighting. Science 306, 1370–1373. 10.1126/science.1102506 15550670

[B6] Anthony K. More A. Zhang X. (2014). Activation of silenced cytokine gene promoters by the synergistic effect of TBP-TALE and VP64-TALE activators. PLoS ONE 9:95790. 10.1371/journal.pone.0095790 24755922 PMC3995891

[B7] Aramaki S. Hatta K. (2006). Visualizing neurons one-by-one *in vivo*: optical dissection and reconstruction of neural networks with reversible fluorescent proteins. Dev. Dyn. 235, 2192–2199. 10.1002/dvdy.20826 16607643

[B8] Arrowsmith C. H. Bountra C. Fish P. V. Lee K. Schapira M. (2012). Epigenetic protein families: a new frontier for drug discovery. Nat. Rev. Drug Discov. 11, 384–400. 10.1038/nrd3674 22498752

[B9] Bachand M. Trent A. M. Bunker B. C. Bachand G. D. (2005). Physical factors affecting kinesin-based transport of synthetic nanoparticle cargo. J. Nanosci. Nanotechnol. 5, 718–722. 10.1166/jnn.2005.112 16010927

[B10] Ballister E. R. Lampson M. A. (2016). Probing mitosis by manipulating the interactions of mitotic regulator proteins using Rapamycin-inducible dimerization. Methods Mol. Biol. 1413, 325–331. 10.1007/978-1-4939-3542-0_20 27193858

[B11] Ballister E. R. Riegman M. Lampson M. A. (2014). Recruitment of Mad1 to metaphase kinetochores is sufficient to reactivate the mitotic checkpoint. J. Cell Biol. 204, 901–908. 10.1083/jcb.201311113 24637323 PMC3998811

[B12] Balzani V. V. Credi A. Raymo F. M. Stoddart J. F. (2000). Artificial molecular machines. Angew. Chem. Int. Ed. Engl. 39, 3348–3391. 10.1002/1521-3773(20001002)39:19<3348::AID-ANIE3348>3.0.CO;2-X 11091368

[B13] Banaszynski L. A. Chen L. Maynard-Smith L. A. Ooi A. G. L. Wandless T. J. (2006). A rapid, reversible, and tunable method to regulate protein function in living cells using synthetic small molecules. Cell 126, 995–1004. 10.1016/j.cell.2006.07.025 16959577 PMC3290523

[B14] Banaszynski L. A. Liu C. W. Wandless T. J. (2005). Characterization of the FKBP-rapamycin-FRB ternary complex. J. Am. Chem. Soc. 127, 4715–4721. 10.1021/ja043277y 15796538

[B15] Barakate A. Stephens J. (2016). An overview of CRISPR-Based tools and their improvements: new opportunities in understanding plant-pathogen interactions for better crop protection. Front. Plant Sci. 7:765. 10.3389/fpls.2016.00765 27313592 PMC4887484

[B16] Bear J. E. Loureiro J. J. Libova I. Fässler R. Wehland J. Gertler F. B. (2000). Negative regulation of fibroblast motility by Ena/VASP proteins. Cell 101, 717–728. 10.1016/S0092-8674(00)80884-3 10892743

[B17] Bertrand E. Chartrand P. Schaefer M. Shenoy S. M. Singer R. H. Long R. M. (1998). Localization of ASH1 mRNA particles in living yeast. Mol. Cell 2, 437–445. 10.1016/S1097-2765(00)80143-4 9809065

[B18] Bikard D. Marraffini L. A. (2013). Control of gene expression by CRISPR-Cas systems. F1000Prime Rep. 5:47. 10.12703/P5-47 24273648 PMC3816762

[B19] Blobel G. Dobberstein B. (1975). Transfer of proteins across membranes. I. Presence of proteolytically processed and unprocessed nascent immunoglobulin light chains on membrane-bound ribosomes of murine myeloma. J. Cell Biol. 67, 835–851. 811671 10.1083/jcb.67.3.835 PMC2111658

[B20] Boch J. Scholze H. Schornack S. Landgraf A. Hahn S. Kay S.. (2009). Breaking the code of DNA binding specificity of TAL-type III effectors. Science 326, 1509–1512. 10.1126/science.1178811 19933107

[B21] Boyden E. S. Zhang F. Bamberg E. Nagel G. Deisseroth K. (2005). Millisecond-timescale, genetically targeted optical control of neural activity. Nat. Neurosci. 8, 1263–1268. 10.1038/nn1525 16116447

[B22] Brouns S. J. J. Jore M. M. Lundgren M. Westra E. R. Slijkhuis R. J. H. Snijders A. P. L.. (2008). Small CRISPR RNAs guide antiviral defense in Prokaryotes. Science 321, 960–964. 10.1126/science.1159689 18703739 PMC5898235

[B23] Buckley C. E. Moore R. E. Reade A. Goldberg A. R. Weiner O. D. Clarke J. D. W. (2016). Reversible optogenetic control of subcellular protein localization in a live Vertebrate Embryo. Dev. Cell 36, 117–126. 10.1016/j.devcel.2015.12.011 26766447 PMC4712025

[B24] Carter C. W. Wolfenden R. (2015). tRNA acceptor stem and anticodon bases form independent codes related to protein folding. Proc. Natl. Acad. Sci. U.S.A. 2015, 201507569. 10.1073/pnas.1507569112 26034281 PMC4475997

[B25] Cermak T. Doyle E. L. Christian M. Wang L. Zhang Y. Schmidt C.. (2011). Efficient design and assembly of custom TALEN and other TAL effector-based constructs for DNA targeting. Nucleic Acids Res. 39, e82–e82. 10.1093/nar/gkr218 21493687 PMC3130291

[B26] Chattopadhyay S. Hung S. C. Stuart A. C. Palmer A. G. III Garcia-Mena J. Das A.. (1995). Interaction between the phage HK022 Nun protein and the nut RNA of phage l. Proc. Natl. Acad. Sci. U.S.A. 92, 12131–12135. 10.1073/pnas.92.26.12131 8618858 PMC40310

[B27] Chen D. Gibson E. S. Kennedy M. J. (2013). A light-triggered protein secretion system. J. Cell Biol. 201, 631–640. 10.1083/jcb.201210119 23671313 PMC3653365

[B28] Chen S. Sanjana N. E. Zheng K. Shalem O. Lee K. Shi X.. (2015). Genome-wide CRISPR Screen in a Mouse Model of Tumor Growth and Metastasis. Cell 160, 1246–1260. 10.1016/j.cell.2015.02.038 25748654 PMC4380877

[B29] Chen X. Wang X. Du Z. Ma Z. Yang Y. (2013). Spatiotemporal control of gene expression in mammalian cells and in mice using the LightOn system, in Current Protocols in Chemical Biology (Hoboken, NJ: John Wiley & Sons, Inc.), 111–129. 10.1002/9780470559277.ch120267 23839993

[B30] Cheng C. Stoddart J. F. (2016). Wholly synthetic molecular machines. Chemphyschem 1780–1793. 10.1002/cphc.201501155 26833859

[B31] Cho H.-S. Kang J. G. Lee J.-H. Lee J.-J. Jeon S. K. Ko J.-H.. (2015). Direct regulation of E-cadherin by targeted histone methylation of TALE-SET fusion protein in cancer cells. Oncotarget 6, 23837–23844. 10.18632/oncotarget.4340 26125227 PMC4695155

[B32] Chow B. Y. Han X. Dobry A. S. Qian X. Chuong A. S. Li M.. (2010). High-performance genetically targetable optical neural silencing by light-driven proton pumps. Nature 463, 98–102. 10.1038/nature08652 20054397 PMC2939492

[B33] Christie J. M. Arvai A. S. Baxter K. J. Heilmann M. Ashley J. Hara A. O.. (2012). Plant UVR8 photoreceptor senses UV-B by Tryptophan-mediated disruption of cross-dimer salt Bridges. 335, 1492–1496. 10.1126/science.1218091.Plant 22323738 PMC3505452

[B34] Christie J. M. Salomon M. Nozue K. Wada M. Briggs W. R. (1999). LOV (light, oxygen, or voltage) domains of the blue-light photoreceptor phototropin (nph1): binding sites for the chromophore flavin mononucleotide. Proc. Natl. Acad. Sci. U.S.A. 96, 8779–8783. 10411952 10.1073/pnas.96.15.8779 PMC17593

[B35] Chung J. Kuo C. J. Crabtree G. R. Blenis J. (1992). Rapamycin-FKBP specifically blocks growth-dependent activation of and signaling by the 70 kd S6 protein kinases. Cell 69, 1227–1236. 1377606 10.1016/0092-8674(92)90643-q

[B36] Collin J. P. Dietrich-Buchecker C. Gaviña P. Jimenez-Molero M. C. Sauvage J. P. (2001). Shuttles and muscles: linear molecular machines based on transition metals. Acc. Chem. Res. 34, 477–487. 10.1021/ar0001766 11412084

[B37] Cong L. Ran F. A. Cox D. Lin S. Barretto R. Habib N.. (2013). Multiplex genome engineering using CRISPR/Cas systems. Science 339, 819–823. 10.1126/science.1231143 23287718 PMC3795411

[B38] Cooper C. Guo J. Yan Y. Chooniedass-Kothari S. Hube F. Hamedani M. K.. (2009). Increasing the relative expression of endogenous non-coding Steroid Receptor RNA Activator (SRA) in human breast cancer cells using modified oligonucleotides. Nucleic Acids Res. 37, 4518–4531. 10.1093/nar/gkp441 19483093 PMC2715257

[B39] Copeland N. G. Jenkins N. A. Court D. L. (2001). Recombineering: a powerful new tool for mouse functional genomics. Nat. Rev. Genet. 2, 769–779. 10.1038/35093556 11584293

[B40] Crefcoeur R. P. Yin R. Ulm R. Halazonetis T. D. (2013). Ultraviolet-B-mediated induction of protein-protein interactions in mammalian cells. Nat. Commun. 4:1779. 10.1038/ncomms2800 23653191

[B41] Crosson S. Rajagopal S. Moffat K. (2003). The LOV domain family: photoresponsive signaling modules coupled to diverse output domains. Biochemistry 42, 2–10. 10.1021/bi026978l 12515534

[B42] Dahlman J. E. Abudayyeh O. O. Joung J. Gootenberg J. S. Zhang F. Konermann S. (2015). Orthogonal gene knockout and activation with a catalytically active Cas9 nuclease. Nat. Biotechnol. 33, 1159–1161. 10.1038/nbt.3390 26436575 PMC4747789

[B43] Daigle N. Ellenberg J. (2007). LambdaN-GFP: an RNA reporter system for live-cell imaging. Nat. Methods 4, 633–636. 10.1038/nmeth1065 17603490

[B44] Delebecque C. J. Lindner A. B. Silver P. A. Aldaye F. A. (2011). Organization of intracellular reactions with rationally designed RNA assemblies. Science 333, 470–474. 10.1126/science.1206938 21700839

[B45] DeRose R. Miyamoto T. Inoue T. (2013). Manipulating signaling at will: chemically-inducible dimerization (CID) techniques resolve problems in cell biology. Pflug. Arch. 465, 409–417. 10.1007/s00424-012-1208-6 23299847 PMC3584178

[B46] Di Ventura B. Kuhlman B. (2016). Go in! Go out! Inducible control of nuclear localization. Curr. Opin. Chem. Biol. 34, 62–71. 10.1016/j.cbpa.2016.06.009 27372352 PMC5107339

[B47] Dohmen R. J. Wu P. Varshavsky A. (1994). Heat-inducible degron: a method for constructing temperature-sensitive mutants. Science 263, 1273–1276. 8122109 10.1126/science.8122109

[B48] Dow L. E. Fisher J. O'Rourke K. P. Muley A. Kastenhuber E. R. Livshits G.. (2015). Inducible *in vivo* genome editing with CRISPR-Cas9. Nat. Biotechnol. 33, 390–394. 10.1038/nbt.3155 25690852 PMC4390466

[B49] Drinnenberg I. A. Fink G. R. Bartel D. P. (2011). Compatibility with killer explains the rise of RNAi-deficient fungi. Science 333:1592. 10.1126/science.1209575 21921191 PMC3790311

[B50] Van Duyne G. D. (2014). Cre Recombinase. Microbiol. Spectr. 2, 1–16. 10.1128/microbiolspec 26104563

[B51] East-Seletsky A. O'Connell M. R. Knight S. C. Burstein D. Cate J. H. D. Tjian R.. (2016). Two distinct RNase activities of CRISPR-C2c2 enable guide-RNA processing and RNA detection. Nature 538, 270–273. 10.1038/nature19802 27669025 PMC5576363

[B52] Espinosa J. M. Cech T. R. Steitz J. A. Lai F. Orom U. A. Cesaroni M.. (2016). Revisiting lncRNAs: how do you know yours is not an eRNA? Mol. Cell 62, 1–2. 10.1016/j.molcel.2016.03.022 27058782

[B53] Etoc F. Lisse D. Bellaiche Y. Piehler J. Coppey M. Dahan M. (2013). Subcellular control of Rac-GTPase signalling by magnetogenetic manipulation inside living cells. Nat. Nanotechnol. 8, 193–198. 10.1038/nnano.2013.23 23455985

[B54] Evers B. Jastrzebski K. Heijmans J. P. M. Grernrum W. Beijersbergen R. L. Bernards R. (2016). CRISPR knockout screening outperforms shRNA and CRISPRi in identifying essential genes. Nat. Biotechnol. 34, 631–633. 10.1038/nbt.3536 27111720

[B55] Fabian M. R. Sonenberg N. Filipowicz W. (2010). Regulation of mRNA translation and stability by microRNAs. Annu. Rev. Biochem. 79, 351–379. 10.1146/annurev-biochem-060308-103103 20533884

[B56] Fagerlund R. D. Staals R. H. J. Fineran P. C. Dy R. Richter C. Salmond G.. (2015). The Cpf1 CRISPR-Cas protein expands genome-editing tools. Genome Biol. 16, 251. 10.1186/s13059-015-0824-9 26578176 PMC4647450

[B57] Fallis A. (2009). An auxin-based degron system for the rapid depletion of proteins in nonplant cells. Nat. Methods 53, 1689–1699. 10.1017/CBO9781107415324.004 19915560

[B58] Farzadfard F. Perli S. D. Lu T. K. (2013). Tunable and multifunctional eukaryotic transcription factors based on CRISPR/Cas. ACS Synth. Biol. 2, 604–613. 10.1021/sb400081r 23977949 PMC3805333

[B59] Feil R. Wagner J. Metzger D. Chambon P. (1997). Regulation of Cre recombinase activity by mutated estrogen receptor ligand-binding domains. Biochem. Biophys. Res. Commun. 237, 752–757. 10.1006/bbrc.1997.7124 9299439

[B60] Feng S. Arnold D. B. (2016). Techniques for studying protein trafficking and molecular motors in neurons. Cytoskeleton (Hoboken) 73, 508–515. 10.1002/cm.21274 26800506 PMC4958032

[B61] Feringa B. L. (2007). The art of building small: from molecular switches to molecular motors. J. Org. Chem. 72, 6635–6652. 10.1021/jo070394d 17629332

[B62] Fischer E. S. Park E. Eck M. J. Thomä N. H. (2016). SPLINTS: Small-molecule protein ligand interface stabilizers. Curr. Opin. Struct. Biol. 37, 115–122. 10.1016/j.sbi.2016.01.004 26829757 PMC4834252

[B63] Friedland A. E. Baral R. Singhal P. Loveluck K. Shen S. Sanchez M.. (2015). Characterization of Staphylococcus aureus Cas9: a smaller Cas9 for all-in-one adeno-associated virus delivery and paired nickase applications. Genome Biol. 16:257. 10.1186/s13059-015-0817-8 26596280 PMC4657203

[B64] Furuta A. Amino M. Yoshio M. Oiwa K. Kojima H. Furuta K. (2017). Creating biomolecular motors based on dynein and actin-binding proteins. Nat. Nanotechnol. 12, 233–237. 10.1038/nnano.2016.238 27842063

[B65] Gao P. Yang H. Rajashankar K. R. Huang Z. Patel D. J. (2016). Type V CRISPR-Cas Cpf1 endonuclease employs a unique mechanism for crRNA-mediated target DNA recognition. Cell Res. 26, 901–913. 10.1038/cr.2016.88 27444870 PMC4973337

[B66] Gasiunas G. Barrangou R. Horvath P. Siksnys V. (2012). Cas9-crRNA ribonucleoprotein complex mediates specific DNA cleavage for adaptive immunity in bacteria. Proc. Natl. Acad. Sci. U.S.A. 109, E2579–E2586. 10.1073/pnas.1208507109 22949671 PMC3465414

[B67] Gasser C. Taiber S. Yeh C.-M. Wittig C. H. Hegemann P. Ryu S.. (2014). Engineering of a red-light-activated human cAMP/cGMP-specific phosphodiesterase. Proc. Natl. Acad. Sci. U.S.A. 111, 8803–8808. 10.1073/pnas.1321600111 24889611 PMC4066486

[B68] Gilbert L. A. Horlbeck M. A. Adamson B. Villalta J. E. Chen Y. Whitehead E. H.. (2014). Genome-scale CRISPR-mediated control of gene repression and activation. Cell 159, 647–661. 10.1016/j.cell.2014.09.029 25307932 PMC4253859

[B69] Gilbert L. A. Larson M. H. Morsut L. Liu Z. Brar G. A. Torres S. E.. (2013). XCRISPR-mediated modular RNA-guided regulation of transcription in eukaryotes. Cell 154, 442–451. 10.1016/j.cell.2013.06.044 23849981 PMC3770145

[B70] Gonzalez B. Schwimmer L. J. Fuller R. P. Ye Y. Asawapornmongkol L. Barbas C. F. (2010). Modular system for the construction of zinc-finger libraries and proteins. Nat. Protoc. 5, 791–810. 10.1038/nprot.2010.34 20360772 PMC2855653

[B71] González F. Zhu Z. Shi Z. D. Lelli K. Verma N. Li Q. V.. (2014). An iCRISPR platform for rapid, multiplexable, and inducible genome editing in human pluripotent stem cells. Cell Stem Cell 15, 215–226. 10.1016/j.stem.2014.05.018 24931489 PMC4127112

[B72] Gonzalez I. Munita R. Agirre E. Dittmer T. A. Gysling K. Misteli T.. (2015). A lncRNA regulates alternative splicing via establishment of a splicing-specific chromatin signature. Nat. Struct. Mol. Biol. 22, 370–376. 10.1038/nsmb.3005 25849144 PMC6322542

[B73] Grizot S. Smith J. Daboussi F. Prieto J. Redondo P. Merino N.. (2009). Efficient targeting of a SCID gene by an engineered single-chain homing endonuclease. Nucleic Acids Res. 37, 5405–5419. 10.1093/nar/gkp548 19584299 PMC2760784

[B74] Guilinger J. P. Thompson D. B. Liu D. R. (2014). Fusion of catalytically inactive Cas9 to FokI nuclease improves the specificity of genome modification. Nat. Biotechnol. 32, 577–582. 10.1038/nbt.2909 24770324 PMC4263420

[B75] Guru A. Post R. J. Ho Y.-Y. Warden M. R. (2015). Making Sense of Optogenetics. Int. J. Neuropsychopharmacol. 18:pyv079. 10.1093/ijnp/pyv079 26209858 PMC4756725

[B76] Haugwitz M. Garachtchenko T. Nourzaie O. Gandlur S. Sagawa H. (2008). Rapid, on-demand protein stabilization and destabilization using the ProteoTuner™ systems. Nat. Methods 5. 10.1038/nmeth.f.223

[B77] Haurwitz R. E. Jinek M. Wiedenheft B. Zhou K. Doudna J. A. (2010). Sequence- and structure-specific RNA processing by a CRISPR endonuclease. Science 329, 1355–1358. 10.1126/science.1192272 20829488 PMC3133607

[B78] Haurwitz R. E. Sternberg S. H. Doudna J. A. (2012). Csy4 relies on an unusual catalytic dyad to position and cleave CRISPR RNA. EMBO J. 31, 2824–2832. 10.1038/emboj.2012.107 22522703 PMC3380207

[B79] Hershko A. Ciechanover A. Heller H. Haas A. L. Rose I. A. (1980). Proposed role of ATP in protein breakdown: conjugation of protein with multiple chains of the polypeptide of ATP-dependent proteolysis. Proc. Natl. Acad. Sci. U.S.A. 77, 1783–1786. 6990414 10.1073/pnas.77.4.1783 PMC348591

[B80] Hilton I. B. M D. A. Vockley C. M. Thakore P. I. Crawford G. E. Reddy T. E.. (2015). Epigenome editing by a {CRISPR-Cas9-based} acetyltransferase activates genes from promoters and enhancers. Nat. Biotechnol. 33, 510–517. 10.1038/nbt.3199 25849900 PMC4430400

[B81] Huala E. Oeller P. W. Liscum E. Han I. S. Larsen E. Briggs W. R. (1997). Arabidopsis NPH1: a protein kinase with a putative redox-sensing domain. Science 278, 2120–2123. 9405347 10.1126/science.278.5346.2120

[B82] Huang J. Makabe K. Biancalana M. Koide A. Koide S. (2009). Structural basis for exquisite specificity of affinity clamps, synthetic binding proteins generated through directed domain-interface evolution. J. Mol. Biol. 392, 1221–1231. 10.1016/j.jmb.2009.07.067 19646997 PMC2748140

[B83] Humphrey J. D. Dufresne E. R. Schwartz M. A. (2014). Mechanotransduction and extracellular matrix homeostasis. Nat. Rev. Mol. Cell Biol. 15, 802–812. 10.1038/nrm3896 25355505 PMC4513363

[B84] Inagaki H. K. Jung Y. Hoopfer E. D. Wong A. M. Mishra N. Lin J. Y.. (2014). Optogenetic control of Drosophila using a red-shifted channelrhodopsin reveals experience-dependent influences on courtship. Nat. Methods 11, 325–332. 10.1038/nmeth.2765 24363022 PMC4151318

[B85] Inobe T. Nozaki M. Nukina N. (2015). Artificial regulation of p53 function by modulating its assembly. Biochem. Biophys. Res. Commun. 467, 322–327. 10.1016/j.bbrc.2015.09.162 26454170

[B86] Iskratsch T. Wolfenson H. Sheetz M. P. (2014). Appreciating force and shape — the rise of mechanotransduction in cell biology. Nat. Rev. Mol. Cell Biol. 15, 825–833. 10.1038/nrm3903 25355507 PMC9339222

[B87] Iwamoto M. Björklund T. Lundberg C. Kirik D. Wandless T. J. (2010). A general chemical method to regulate protein stability in the mammalian central nervous system. Chem. Biol. 17, 981–988. 10.1016/j.chembiol.2010.07.009 20851347 PMC2943492

[B88] Jacques S. L. (2013). Corrigendum: optical properties of biological tissues: a review. Phys. Med. Biol. 58, 5007–5008. 10.1088/0031-9155/58/14/5007 23666068

[B89] Jansen V. Alvarez L. Balbach M. Strünker T. Hegemann P. Kaupp U. B.. (2015). Controlling fertilization and cAMP signaling in sperm by optogenetics. Elife 4:e05161. 10.7554/eLife.05161 25601414 PMC4298566

[B90] Jentzsch K. Wirtz A. Circolone F. Drepper T. Losi A. Gärtner W.. (2009). Mutual exchange of kinetic properties by extended mutagenesis in two short LOV domain proteins from *Pseudomonas putida*. Biochemistry 48, 10321–10333. 10.1021/bi901115z 19772355

[B91] Jin C. Qin T. Barton M. C. Jelinek J. Issa J. P. J. (2015). Minimal role of base excision repair in TET-induced global DNA demethylation in HEK293T cells. Epigenetics 10, 1006–1013. 10.1080/15592294.2015.1091145 26440216 PMC4844212

[B92] Jinek M. Chylinski K. Fonfara I. Hauer M. Doudna J. A. Charpentier E. (2012). A programmable dual-RNA-guided DNA endonuclease in adaptive bacterial immunity. Science 337, 816–821. 10.1126/science.1225829 22745249 PMC6286148

[B93] Jullien N. (2003). Regulation of Cre recombinase by ligand-induced complementation of inactive fragments. Nucleic Acids Res. 31, 131e–131. 10.1093/nar/gng131 14576331 PMC275488

[B94] Karimova M. Splith V. Karpinski J. Pisabarro M. T. Buchholz F. (2016). Discovery of Nigri/nox and Panto/pox site-specific recombinase systems facilitates advanced genome engineering. Sci. Rep. 6:30130. 10.1038/srep30130 27444945 PMC4957104

[B95] Katz Z. B. Wells A. L. Park H. Y. Wu B. Shenoy S. M. Singer R. H. (2012). -Actin mRNA compartmentalization enhances focal adhesion stability and directs cell migration. Genes Dev. 26, 1885–1890. 10.1101/gad.190413.112 22948660 PMC3435492

[B96] Kawano F. Okazaki R. Yazawa M. Sato M. (2016). A photoactivatable Cre-*loxP* recombination system for optogenetic genome engineering. Nat. Chem. Biol. 12, 1059–1064. 10.1038/nchembio.2205 27723747

[B97] Kawano F. Suzuki H. Furuya A. Sato M. (2015). Engineered pairs of distinct photoswitches for optogenetic control of cellular proteins. Nat. Commun. 6:6256. 10.1038/ncomms7256 25708714

[B98] Kearns N. A. Pham H. Tabak B. Genga R. M. Silverstein N. J. Garber M.. (2015). Functional annotation of native enhancers with a Cas9-histone demethylase fusion. Nat. Methods 12, 401–403. 10.1038/nmeth.3325 25775043 PMC4414811

[B99] Kelwick R. MacDonald J. T. Webb A. J. Freemont P. (2014). Developments in the tools and methodologies of synthetic biology. Front. Bioeng. Biotechnol. 2:60. 10.3389/fbioe.2014.00060 25505788 PMC4244866

[B100] Kennedy M. B. (1995). Origin of PDZ (DHR, GLGF) domains. Trends Biochem. Sci. 20:350. 10.1016/S0968-0004(00)89074-X 7482701

[B101] Kennedy M. J. Hughes R. M. Peteya L. A. Schwartz J. W. Ehlers M. D. Tucker C. L. (2010). Rapid blue-light-mediated induction of protein interactions in living cells. Nat. Methods 7, 973–975. 10.1038/nmeth.1524 21037589 PMC3059133

[B102] Keung A. J. Khalil A. S. (2016). A unifying model of epigenetic regulation. Science 351, 661–662. 10.1126/science.aaf1647 26912843 PMC6124315

[B103] Kim B. Lin M. Z. (2013). Optobiology: optical control of biological processes via protein engineering. Biochem. Soc. Trans. 41, 1183–1188. 10.1042/BST20130150 24059506 PMC4076147

[B104] Kim D. Kim J. Hur J. K. Been K. W. Yoon S. Kim J.-S. (2016). Genome-wide analysis reveals specificities of Cpf1 endonucleases in human cells. Nat. Biotechnol. 34, 863–868. 10.1038/nbt.3609 27272384

[B105] Kim D.-S. Gusti V. Pillai S. G. Gaur R. K. (2005). An artificial riboswitch for controlling pre-mRNA splicing. RNA 11, 1667–1677. 10.1261/rna.2162205 16244133 PMC1370853

[B106] Kim H. Kim J. S. (2014). A guide to genome engineering with programmable nucleases. Nat. Rev. Genet. 15, 321–334. 10.1038/nrg3686 24690881

[B107] Kim Y. G. Cha J. Chandrasegaran S. (1996). Hybrid restriction enzymes: zinc finger fusions to Fok I cleavage domain. Proc. Natl. Acad. Sci. U.S.A. 93, 1156–1160. 10.1073/pnas.93.3.1156 8577732 PMC40048

[B108] Kleinstiver B. P. Tsai S. Q. Prew M. S. Nguyen N. T. Welch M. M. Lopez J. M.. (2016). Genome-wide specificities of CRISPR-Cas Cpf1 nucleases in human cells. Nat. Biotechnol. 34, 869–874. 10.1038/nbt.3620 27347757 PMC4980201

[B109] Konermann S. Brigham M. D. Trevino A. E. Hsu P. D. Heidenreich M. Cong L.. (2013). Optical control of mammalian endogenous transcription and epigenetic states. Nature 500, 472–476. 10.1038/nature12466 23877069 PMC3856241

[B110] Kooistra S. M. Helin K. (2012). Molecular mechanisms and potential functions of histone demethylases. Nat. Rev. Mol. Cell Biol. 13, 297–311. 10.1038/nrm3327 22473470

[B111] Kopan R. (2002). Notch: a membrane-bound transcription factor. J. Cell Sci. 115, 1095–1097. Available online at: http://jcs.biologists.org/content/115/6/1095.long 11884509 10.1242/jcs.115.6.1095

[B112] Kornienko A. E. Guenzl P. M. Barlow D. P. Pauler F. M. (2013). Gene regulation by the act of long non-coding RNA transcription. BMC Biol. 11:59. 10.1186/1741-7007-11-59 23721193 PMC3668284

[B113] Larson M. H. Gilbert L. A. Wang X. Lim W. A. Weissman J. S. Qi L. S. (2013). CRISPR interference (CRISPRi) for sequence-specific control of gene expression. Nat. Protoc. 8, 2180–2196. 10.1038/nprot.2013.132 24136345 PMC3922765

[B114] Laufer B. I. Singh S. M. Groote M. Verschure P. Rots M. Jurkowski T.. (2015). Strategies for precision modulation of gene expression by epigenome editing: an overview. Epigenet. Chromatin 8:34. 10.1186/s13072-015-0023-7 26388942 PMC4574080

[B115] Lee S. Kopp F. Chang T.-C. Sataluri A. Chen B. Sivakumar S.. (2016). Noncoding RNA NORAD regulates genomic stability by sequestering PUMILIO proteins. Cell 164, 69–80. 10.1016/j.cell.2015.12.017 26724866 PMC4715682

[B116] Levskaya A. Weiner O. D. Lim W. A. Voigt C. A. (2009). Spatiotemporal control of cell signalling using a light- switchable protein interaction. Nature 461, 997–1001. 10.1038/nature08446 19749742 PMC2989900

[B117] Lévy F. Johnston J. A. Varshavsky A. (1999). Analysis of a conditional degradation signal in yeast and mammalian cells. Eur. J. Biochem. 259, 244–252. 9914499 10.1046/j.1432-1327.1999.00024.x

[B118] Li J. Kim S. G. Blenis J. (2014). Rapamycin: one drug, many effects. Cell Metab. 19, 373–379. 10.1016/j.cmet.2014.01.001 24508508 PMC3972801

[B119] Li W. Teng F. Li T. Zhou Q. (2013). Simultaneous generation and germline transmission of multiple gene mutations in rat using CRISPR-Cas systems. Nat. Biotechnol. 31, 684–686. 10.1038/nbt.2652 23929337

[B120] Lim F. Downey T. P. Peabody D. S. (2001). Translational repression and specific RNA Binding by the Coat Protein of the Pseudomonas Phage PP7. J. Biol. Chem. 276, 22507–22513. 10.1074/jbc.M102411200 11306589

[B121] Lim F. Peabody D. S. (2002). RNA recognition site of PP7 coat protein. Nucleic Acids Res. 30, 4138–4144. 10.1093/nar/gkf552 12364592 PMC140551

[B122] Lin J. Y. Knutsen P. M. Muller A. Kleinfeld D. Tsien R. Y. (2013). ReaChR: a red-shifted variant of channelrhodopsin enables deep transcranial optogenetic excitation. Nat. Neurosci. 16, 1499–1508. 10.1038/nn.3502 23995068 PMC3793847

[B123] Liu H. Liu B. Zhao C. Pepper M. Lin C. (2011). The action mechanisms of plant cryptochromes. Trends Plant Sci. 16, 684–691. 10.1016/j.tplants.2011.09.002 21983106 PMC3277817

[B124] Liu H. Wang Q. Liu Y. Zhao X. Imaizumi T. Somers D. E.. (2013). Arabidopsis CRY2 and ZTL mediate blue-light regulation of the transcription factor CIB1 by distinct mechanisms. Proc. Natl. Acad. Sci. U.S.A. 110, 17582–17587. 10.1073/pnas.1308987110 24101505 PMC3808666

[B125] Liu H. Yu X. Li K. Klejnot J. Yang H. Lisiero D.. (2008). Photoexcited CRY2 interacts with CIB1 to regulate transcription and floral initiation in *Arabidopsis*. Science 322, 1535–1539. 10.1126/science.1163927 18988809

[B126] Liu Z. Liu Y. Chang Y. Seyf H. R. Henry A. Mattheyses A. L.. (2016). Nanoscale optomechanical actuators for controlling mechanotransduction in living cells. Nat. Methods 13, 143–146. 10.1038/nmeth.3689 26657558 PMC4732909

[B127] Lokhandwala J. Silverman Y. De La Vega R. I. Hopkins H. C. Britton C. W. Rodriguez-Iglesias A.. (2016). A native threonine coordinates ordered water to tune light-oxygen-voltage (LOV) domain photocycle kinetics and osmotic stress signaling in trichoderma reesei ENVOY. J. Biol. Chem. 291, 14839–14850. 10.1074/jbc.M116.731448 27226624 PMC4938200

[B128] Losi A. Polverini E. Quest B. Gärtner W. (2002). First evidence for phototropin-related blue-light receptors in prokaryotes. Biophys. J. 82, 2627–2634. 10.1016/S0006-3495(02)75604-X 11964249 PMC1302051

[B129] Ma Y. Zhang L. Huang X. (2014). Genome modification by CRISPR/Cas9. FEBS J. 281, 5186–5193. 10.1111/febs.13110 25315507

[B130] Maeder M. L. Angstman J. F. Richardson M. E. Linder S. J. Cascio V. M. Tsai S. Q.. (2013). Targeted DNA demethylation and activation of endogenous genes using programmable TALE-TET1 fusion proteins. Nat. Biotechnol. 31, 1137–1142. 10.1038/nbt.2726 24108092 PMC3858462

[B131] Maeder M. L. Thibodeau-Beganny S. Sander J. D. Voytas D. F. Joung J. K. (2009). Oligomerized pool engineering (OPEN): an “open-source” protocol for making customized zinc-finger arrays. Nat. Protoc. 4, 1471–1501. 10.1038/nprot.2009.98 19798082 PMC2858690

[B132] Mali P. Yang L. Esvelt K. M. Aach J. Guell M. DiCarlo J. E.. (2013). RNA-guided human genome engineering via Cas9. Science 339, 823–826. 10.1126/science.1232033 23287722 PMC3712628

[B133] Mali Prashant E. K. C. G.. (2014). Cas9 as a versatile tool for engeneering biology. NIH Public Access 10, 957–963. 10.1038/nmeth.2649.Cas9 24076990 PMC4051438

[B134] Miller J. C. Tan S. Qiao G. Barlow K. A. Wang J. Xia D. F.. (2011). A TALE nuclease architecture for efficient genome editing. Nat. Biotechnol. 29, 143–148. 10.1038/nbt.1755 21179091

[B135] Mojica F. J. M. Díez-Villaseñor C. García-Martínez J. Almendros C. (2009). Short motif sequences determine the targets of the prokaryotic CRISPR defence system. Microbiology 155, 733–740. 10.1099/mic.0.023960-0 19246744

[B136] Moras D. Poterszman A. (1995). RNA-Protein Interactions: diverse modes of recognition. Curr. Biol. 5, 249–251. 10.1016/S0960-9822(95)00051-0 7540101

[B137] Morawska M. Ulrich H. D. (2013). An expanded tool kit for the auxin-inducible degron system in budding yeast. Yeast 30, 341–351. 10.1002/yea.2967 23836714 PMC4171812

[B138] Morsut L. Roybal K. T. Xiong X. Gordley R. M. Coyle S. M. Thomson M.. (2016). Engineering customized cell sensing and response behaviors using synthetic notch receptors. Cell 164, 780–791. 10.1016/j.cell.2016.01.012 26830878 PMC4752866

[B139] Moscou M. J. Bogdanove A. J. (2009). A simple cipher governs DNA recognition by TAL effectors. Science 326, 1501. 10.1126/science.1178817 19933106

[B140] Müller K. Engesser R. Schulz S. Steinberg T. Tomakidi P. Weber C. C.. (2013). Multi-chromatic control of mammalian gene expression and signaling. Nucleic Acids Res. 41:e124. 10.1093/nar/gkt340 23625964 PMC3695509

[B141] Murata Y. Wharton R. P. (1995). Binding of pumilio to maternal hunchback mRNA is required for posterior patterning in drosophila embryos. Cell 80, 747–756. 10.1016/0092-8674(95)90353-4 7889568

[B142] Mussolino C. Cathomen T. (2012). TALE nucleases: tailored genome engineering made easy. Curr. Opin. Biotechnol. 23, 644–650. 10.1016/j.copbio.2012.01.013 22342754

[B143] Mussolino C. Morbitzer R. Lütge F. Dannemann N. Lahaye T. Cathomen T. (2011). A novel TALE nuclease scaffold enables high genome editing activity in combination with low toxicity. Nucleic Acids Res. 39, 9283–9293. 10.1093/nar/gkr597 21813459 PMC3241638

[B144] Nagel G. Szellas T. Huhn W. Kateriya S. Adeishvili N. Berthold P.. (2003). Channelrhodopsin-2, a directly light-gated cation-selective membrane channel. Proc. Natl. Acad. Sci. U.S.A. 100, 13940–13945. 10.1073/pnas.1936192100 14615590 PMC283525

[B145] Nelson B. R. Makarewich C. A. Anderson D. M. Winders B. R. Troupes C. D. Wu F.. (2016). A peptide encoded by a transcript annotated as long noncoding RNA enhances SERCA activity in muscle. Science 351, 271–275. 10.1126/science.aad4076 26816378 PMC4892890

[B146] Nguyen M. K. Kim C. Y. Kim J. M. Park B. O. Lee S. Park H.. (2016). Optogenetic oligomerization of Rab GTPases regulates intracellular membrane trafficking. Nat. Chem. Biol. 12, 431–436. 10.1038/nchembio.2064 27065232

[B147] Ni M. Tepperman J. M. Quail P. H. (1999). Binding of phytochrome B to its nuclear signalling partner PIF3 is reversibly induced by light. Nature 400, 781–784. 10.1038/23500 10466729

[B148] Nihongaki Y. Kawano F. Nakajima T. Sato M. (2015). Photoactivatable CRISPR-Cas9 for optogenetic genome editing. Nat. Biotechnol. 33, 755–760. 10.1038/nbt.3245 26076431

[B149] Niopek D. Benzinger D. Roensch J. Draebing T. Wehler P. Eils R.. (2014). Engineering light-inducible nuclear localization signals for precise spatiotemporal control of protein dynamics in living cells. Nat. Commun. 5, 4404. 10.1038/ncomms5404 25019686 PMC4104460

[B150] Niopek D. Wehler P. Roensch J. Eils R. Di Ventura B. (2016). Optogenetic control of nuclear protein export. Nat. Commun. 7:10624. 10.1038/ncomms10624 26853913 PMC4748110

[B151] Nishimura K. Fukagawa T. Takisawa H. Kakimoto T. Kanemaki M. (2009). An auxin-based degron system for the rapid depletion of proteins in nonplant cells. Nat. Methods 6, 917–922. 10.1038/nmeth.1401 19915560

[B152] Nissim L. Perli S. D. Fridkin A. Perez-Pinera P. Lu T. K. (2014). Multiplexed and programmable regulation of gene networks with an integrated RNA and CRISPR/Cas toolkit in human cells. Mol. Cell 54, 698–710. 10.1016/j.molcel.2014.04.022 24837679 PMC4077618

[B153] Niu Y. Shen B. Cui Y. Chen Y. Wang J. Wang L.. (2014). Generation of gene-modified cynomolgus monkey via Cas9/RNA-mediated gene targeting in one-cell embryos. Cell 156, 836–843. 10.1016/j.cell.2014.01.027 24486104

[B154] Oubridge C. Ito N. Evans P. R. Teo C. H. Nagai K. (1994). Crystal structure at 1.92 A resolution of the RNA-binding domain of the U1A spliceosomal protein complexed with an RNA hairpin. Nature 372, 432–438. 10.1038/372432a0 7984237

[B155] Pal S. Tyler J. K. (2016). Epigenetics and aging. Sci. Adv. 2:e1600584. 10.1126/sciadv.1600584 27482540 PMC4966880

[B156] Park S.-J. Gazzola M. Park K. S. Park S. Di Santo V. Blevins E. L.. (2016). Phototactic guidance of a tissue-engineered soft-robotic ray. Science 353, 158–162. 10.1126/science.aaf4292 27387948 PMC5526330

[B157] Potorac I. Rivero-M A. Trehan A. Kiełbus M. Jozwiak K. Pralong F.. (2016). A vital region for human glycoprotein hormone trafficking revealed by an LHB mutation. J. Endocrinol. 231, 197–207. 10.1530/JOE-16-0384 27656125

[B158] Przybilski R. Richter C. Gristwood T. Clulow J. S. Vercoe R. B. Fineran P. C. (2011). Csy4 is responsible for CRISPR RNA processing in *Pectobacterium atrosepticum*. RNA Biol. 8, 517–528. 10.4161/rna.8.3.15190 21519197

[B159] Putyrski M. Schultz C. (2012). Protein translocation as a tool: the current rapamycin story. FEBS Lett. 586, 2097–2105. 10.1016/j.febslet.2012.04.061 22584056

[B160] Qi L. S. Larson M. H. Gilbert L. A. Doudna J. A. Weissman J. S. Arkin A. P.. (2013). Repurposing CRISPR as an RNA-Guided platform for sequence- specific control of gene expression. Cell 152, 1173–1183. 10.1016/j.cell.2013.02.022.Repurposing 23452860 PMC3664290

[B161] Qi W. Zhu T. Tian Z. Li C. Zhang W. Song R. (2016). High-efficiency {CRISPR/Cas9} multiplex gene editing using the glycine {tRNA-processing} system-based strategy in maize. BMC Biotechnol. 16:58. 10.1186/s12896-016-0289-2 27515683 PMC4982333

[B162] Qin W. Liang F. Feng Y. Bai H. Yan R. Li S.. (2015). Expansion of CRISPR/Cas9 genome targeting sites in zebrafish by Csy4-based RNA processing. Cell Res. 25, 1074–1077. 10.1038/cr.2015.95 26238401 PMC4559817

[B163] Quintino L. Manfré G. Wettergren E. E. Namislo A. Isaksson C. Lundberg C. (2013). Functional neuroprotection and efficient regulation of GDNF using destabilizing domains in a rodent model of Parkinson's disease. Mol. Ther. 21, 2169–2180. 10.1038/mt.2013.169 23881415 PMC3863791

[B164] Rein M. L. Deussing J. M. (2012). The optogenetic (r)evolution. Mol. Genet. Genomics 287, 95–109. 10.1007/s00438-011-0663-7 22183142 PMC3266495

[B165] Robinson M. S. Sahlender D. A. Foster S. D. (2010). Rapid inactivation of proteins by rapamycin-induced rerouting to mitochondria. Dev. Cell 18, 324–331. 10.1016/j.devcel.2009.12.015 20159602 PMC2845799

[B166] Rodriguez-Fraticelli A. E. Vergarajauregui S. Eastburn D. J. Datta A. Alonso M. A. Mostov K.. (2010). The Cdc42 GEF Intersectin 2 controls mitotic spindle orientation to form the lumen during epithelial morphogenesis. J. Cell Biol. 189, 725–738. 10.1083/jcb.201002047 20479469 PMC2872911

[B167] Romaniuk P. J. Lowary P. Wu H. N. Stormo G. Uhlenbeck O. C. (1987). RNA binding site of R17 coat protein. Biochemistry 26, 1563–1568. 10.1021/bi00380a011 3297131

[B168] Root C. M. Denny C. A. Hen R. Axel R. (2014). The participation of cortical amygdala in innate, odour-driven behaviour. Nature 515, 269–273. 10.1038/nature13897 25383519 PMC4231015

[B169] Rumnieks J. Tars K. (2014). Crystal structure of the bacteriophage q?? coat protein in complex with the rna operator of the replicase gene. J. Mol. Biol. 426, 1039–1049. 10.1016/j.jmb.2013.08.025 24035813

[B170] Sachdeva G. Garg A. Godding D. Way J. C. Silver P. A. (2014). *In vivo* co-localization of enzymes on RNA scaffolds increases metabolic production in a geometrically dependent manner. Nucleic Acids Res. 42, 9493–9503. 10.1093/nar/gku617 25034694 PMC4132732

[B171] Sander J. D. Joung J. K. (2014). CRISPR-Cas systems for genome editing, regulation and targeting. Nat. Biotechnol. 32, 347–355. 10.1038/nbt.2842.CRISPR-Cas 24584096 PMC4022601

[B172] Sauer B. McDermott J. (2004). DNA recombination with a heterospecific Cre homolog identified from comparison of the pac-c1 regions of P1-related phages. Nucleic Acids Res. 32, 6086–6095. 10.1093/nar/gkh941 15550568 PMC534624

[B173] Schmid-Burgk J. L. Schmidt T. Kaiser V. Höning K. Hornung V. (2013). A ligation-independent cloning technique for high-throughput assembly of transcription activator–like effector genes. Nat. Biotechnol. 31, 76–81. 10.1038/nbt.2460 23242165 PMC4142318

[B174] Shechner D. M. Hacisuleyman E. Younger S. T. Rinn J. L. (2015). Multiplexable, locus-specific targeting of long RNAs with CRISPR-Display. Nat. Methods 12, 664–670. 10.1038/nmeth.3433 26030444 PMC4821475

[B175] Shmakov S. Abudayyeh O. O. Makarova K. S. Wolf Y. I. Gootenberg J. S. Semenova E.. (2015). Discovery and Functional Characterization of Diverse Class 2 CRISPR-Cas Systems. Mol. Cell 60, 385–397. 10.1016/j.molcel.2015.10.008 26593719 PMC4660269

[B176] Sigal N. H. Dumont F. J. (1992). Cyclosporin A, FK-506, and Rapamycin: pharmacologic probes of lymphocyte signal transduction. Annu. Rev. Immunol. 10, 519–560. 10.1146/annurev.iy.10.040192.002511 1375473

[B177] Skelton N. J. Koehler M. F. T. Zobel K. Wong W. L. Yeh S. Pisabarro M. T.. (2003). Origins of PDZ domain ligand specificity. Structure determination and mutagenesis of the erbin PDZ domain. J. Biol. Chem. 278, 7645–7654. 10.1074/jbc.M209751200 12446668

[B178] Sternberg S. H. Haurwitz R. E. Doudna J. A. (2012). Mechanism of substrate selection by a highly specific CRISPR endoribonuclease. RNA 18, 661–672. 10.1261/rna.030882.111 22345129 PMC3312554

[B179] Stierl M. Stumpf P. Udwari D. Gueta R. Hagedorn R. Losi A.. (2011). Light modulation of cellular cAMP by a small bacterial photoactivated Adenylyl Cyclase, bPAC, of the Soil Bacterium Beggiatoa. J. Biol. Chem. 286, 1181–1188. 10.1074/jbc.M110.185496 21030594 PMC3020725

[B180] Strickland D. Lin Y. Wagner E. Hope C. M. Zayner J. Antoniou C.. (2012). TULIPs: tunable, light-controlled interacting protein tags for cell biology. Nat. Methods 9, 379–384. 10.1038/nmeth.1904 22388287 PMC3444151

[B181] Tai K. Quintino L. Isaksson C. Gussing F. Lundberg C. (2012). Destabilizing domains mediate reversible transgene expression in the brain. PLoS ONE 7:e46269. 10.1371/journal.pone.0046269 23029456 PMC3460874

[B182] Tanenbaum M. E. Gilbert L. A. Qi L. S. Weissman J. S. Vale R. D. (2014). A protein-tagging system for signal amplification in gene expression and fluorescence imaging. Cell 159, 635–646. 10.1016/j.cell.2014.09.039 25307933 PMC4252608

[B183] Taslimi A. Zoltowski B. Miranda J. G. Pathak G. P. Hughes R. M. Tucker C. L. (2016). Optimized second-generation CRY2–CIB dimerizers and photoactivatable Cre recombinase. Nat. Chem. Biol. 12, 1–8. 10.1038/nchembio.2063 27065233 PMC4871718

[B184] Thakore P. I. MD'lppolito A. Song L. Safi A. Shivakumar N. K. Kabadi A. M.. (2015). Highly specific epigenome editing by {CRISPR-Cas9} repressors for silencing of distal regulatory elements. Nat. Methods 12, 1143–1149. 10.1038/nmeth.3630 26501517 PMC4666778

[B185] Thompson K. M. Syrett H. A. Knudsen S. M. Ellington A. D. (2002). Group I aptazymes as genetic regulatory switches. BMC Biotechnol. 2:21. 10.1186/1472-6750-2-21 12466025 PMC139998

[B186] Tischer D. Weiner O. D. (2014). Illuminating cell signalling with optogenetic tools. Nat. Rev. Mol. Cell Biol. 15, 551–558. 10.1038/nrm3837 25027655 PMC4145075

[B187] Tóth E. Weinhardt N. Bencsura P. Huszár K. Kulcsár P. I. Tálas A.. (2016). Cpf1 nucleases demonstrate robust activity to induce DNA modification by exploiting homology directed repair pathways in mammalian cells. Biol. Direct 11:46. 10.1186/s13062-016-0147-0 27630115 PMC5024423

[B188] Tsai S. Q. Wyvekens N. Khayter C. Foden J. A. Thapar V. Reyon D.. (2014). Dimeric CRISPR RNA-guided FokI nucleases for highly specific genome editing. Nat. Biotechnol. 32, 569–576. 10.1038/nbt.2908 24770325 PMC4090141

[B189] Urnov F. D. Rebar E. J. Holmes M. C. Zhang H. S. Gregory P. D. (2010). Genome editing with engineered zinc finger nucleases. Nat. Rev. Genet. 11, 636–646. 10.1038/nrg2842 20717154

[B190] van Bergeijk P. Adrian M. Hoogenraad C. C. Kapitein L. C. (2015). Optogenetic control of organelle transport and positioning. Nature 518, 111–114. 10.1038/nature14128 25561173 PMC5063096

[B191] Van Gilst M. R. Rees W. A. Das A. Von Hippel P. H. (1997). Complexes of N antitermination protein of phage lambda with specific and nonspecific RNA target sites on the nascent transcript. Biochemistry 36, 1514–1524. 10.1021/bi961920q 9063900

[B192] Vojta A. Dobrinic P. Tadic V. Bockor L. Korac P. Julg B.. (2016). Repurposing the CRISPR-Cas9 system for targeted DNA methylation. Nucleic Acids Res. 44, 5615–5628. 10.1093/nar/gkw159 26969735 PMC4937303

[B193] Wang F. Qi L. S. (2016). Applications of CRISPR genome engineering in cell biology. Trends Cell Biol. 26, 875–888. 10.1016/j.tcb.2016.08.004 27599850 PMC5077632

[B194] Wang H. Vilela M. Winkler A. Tarnawski M. Schlichting I. Yumerefendi H.. (2016). LOVTRAP: an optogenetic system for photoinduced protein dissociation. Nat. Methods 13, 755–758. 10.1038/nmeth.3926 27427858 PMC5137947

[B195] Wang H. Yang H. Shivalila C. S. Dawlaty M. M. Cheng A. W. Zhang F.. (2013). One-step generation of mice carrying mutations in multiple genes by CRISPR/Cas-mediated genome engineering. Cell 153, 910–918. 10.1016/j.cell.2013.04.025 23643243 PMC3969854

[B196] Wang X. Chen X. Yang Y. (2012). Spatiotemporal control of gene expression by a light-switchable transgene system. Nat. Methods 9, 266–269. 10.1038/nmeth.1892 22327833

[B197] Wehler P. Niopek D. Eils R. Di Ventura B. (2016). Optogenetic control of nuclear protein import in living cells using Light-Inducible Nuclear Localization Signals (LINuS). Curr. Protoc. Chem. Biol. 8, 131–145. 10.1002/cpch.4 27258691

[B198] Wesley R. Browne B. L. F. (2006). Making molecular machines work. Nat. Nanotechnol. 25–35. 10.1038/nnano.2006.45 18654138

[B199] Wharton R. P. Struhl G. (1991). RNA regulatory elements mediate control of Drosophila body pattern by the posterior morphogen nanos. Cell 67, 955–967. 10.1016/0092-8674(91)90368-9 1720354

[B200] Winters M. J. Lamson R. E. Nakanishi H. Neiman A. M. Pryciak P. M. (2005). A Membrane Binding Domain in the Ste5 Scaffold Synergizes with Gβγ Binding to Control Localization and Signaling in Pheromone Response. Mol. Cell 20, 21–32. 10.1016/j.molcel.2005.08.020 16209942

[B201] Wolff S. B. E. Gründemann J. Tovote P. Krabbe S. Jacobson G. A. Müller C.. (2014). Amygdala interneuron subtypes control fear learning through disinhibition. Nature 509, 453–458. 10.1038/nature13258 24814341

[B202] Wood L. Booth D. G. Vargiu G. Ohta S. deLima Alves F. Samejima K.. (2016). Auxin/AID versus conventional knockouts: distinguishing the roles of CENP-T/W in mitotic kinetochore assembly and stability. Open Biol. 6:150230. 10.1098/rsob.150230 26791246 PMC4736828

[B203] Wright A. V. Nuñez J. K. Doudna J. A. (2016). Biology and applications of CRISPR systems: harnessing nature's toolbox for genome engineering. Cell 164, 29–44. 10.1016/j.cell.2015.12.035 26771484

[B204] Wu D. Hu Q. Yan Z. Chen W. Yan C. Huang X.. (2012). Structural basis of ultraviolet-B perception by UVR8. Nature 484, 214–219. 10.1038/nature10931 22388820

[B205] Wu Y. I. Frey D. Lungu O. I. Jaehrig A. Schlichting I. Kuhlman B.. (2009). A genetically encoded photoactivatable Rac controls the motility of living cells. Nature 461, 104–108. 10.1038/nature08241 19693014 PMC2766670

[B206] Xie K. Minkenberg B. Yang Y. (2015). Boosting CRISPR/Cas9 multiplex editing capability with the endogenous tRNA-processing system. Proc. Natl. Acad. Sci. U.S.A. 112, 3570–3575. 10.1073/pnas.1420294112 25733849 PMC4371917

[B207] Xu L. Zhao L. Gao Y. Xu J. Han R. (2016). Empower multiplex cell and tissue-specific CRISPR-mediated gene manipulation with self-cleaving ribozymes and tRNA. Nucleic Acids Res. [Epub ahead of print]. 10.1093/nar/gkw1048 27799472 PMC5389707

[B208] Xu X. Tao Y. Gao X. Zhang L. Li X. Zou W.. (2016). A CRISPR-based approach for targeted DNA demethylation. Cell Discov. 2, 16009. 10.1038/celldisc.2016.9 27462456 PMC4853773

[B209] Yang Y. Wen L. Zhu H. (2015). Unveiling the hidden function of long non-coding RNA by identifying its major partner-protein. Cell Biosci. 5:59. 10.1186/s13578-015-0050-x 26500759 PMC4618879

[B210] Yeh B. J. Rutigliano R. J. Deb A. Bar-Sagi D. Lim W. A. (2007). Rewiring cellular morphology pathways with synthetic guanine nucleotide exchange factors. Nature 447, 596–600. 10.1038/nature05851 17515921

[B211] Yen L. Svendsen J. Lee J.-S. Gray J. T. Magnier M. Baba T.. (2004). Exogenous control of mammalian gene expression through modulation of RNA self-cleavage. Nature 431, 471–476. 10.1038/nature02844 15386015

[B212] Yoshioka S. Fujii W. Ogawa T. Sugiura K. Naito K. (2015). Development of a mono-promoter-driven CRISPR/Cas9 system in mammalian cells. Sci. Rep. 5:18341. 10.1038/srep18341 26669567 PMC4680873

[B213] Young D. D. Garner R. A. Yoder J. A. Deiters A. (2009). Light-activation of gene function in mammalian cells *via* ribozymes. Chem. Commun. 1, 568–570. 10.1039/b819375d PMC3702056 19283293

[B214] Yumerefendi H. Lerner A. M. Zimmerman S. P. Hahn K. Bear J. E. Strahl B. D.. (2016). Light-induced nuclear export reveals rapid dynamics of epigenetic modifications. Nat. Chem. Biol. 12, 399–401. 10.1038/pj.2016.37 27089030 PMC4888063

[B215] Zalatan J. G. Lee M. E. Almeida R. Gilbert L. A. Whitehead E. H. La Russa M.. (2015). Engineering complex synthetic transcriptional programs with CRISPR RNA scaffolds. Cell 160, 339–350. 10.1016/j.cell.2014.11.052 25533786 PMC4297522

[B216] Zetsche B. Gootenberg J. S. Abudayyeh O. O. Slaymaker I. M. Makarova K. S. Essletzbichler P.. (2015a). Cpf1 is a single RNA-guided endonuclease of a class 2 CRISPR-Cas system. Cell 163, 759–771. 10.1016/j.cell.2015.09.038 26422227 PMC4638220

[B217] Zetsche B. Volz S. E. Zhang F. (2015b). A split-Cas9 architecture for inducible genome editing and transcription modulation. Nat. Biotechnol. 33, 139–142. 10.1038/nbt.3149 25643054 PMC4503468

[B218] Zhang F. Wang L.-P. Brauner M. Liewald J. F. Kay K. Watzke N.. (2007). Multimodal fast optical interrogation of neural circuitry. Nature 446, 633–639. 10.1038/nature05744 17410168

[B219] Zhang K. Cui B. (2015). Optogenetic control of intracellular signaling pathways. Trends Biotechnol. 33, 92–100. 10.1016/j.tibtech.2014.11.007 25529484 PMC4308517

[B220] Zhang L. Ward J. D. Cheng Z. Dernburg A. F. (2015). The auxin-inducible degradation (AID) system enables versatile conditional protein depletion in *C. elegans*. Development 142, 4374–4384. 10.1242/dev.129635 26552885 PMC4689222

[B221] Zhao X.-Y. Lin J. D. (2015). Long noncoding RNAs: a new regulatory code in metabolic control. Trends Biochem. Sci. 40, 586–596. 10.1016/j.tibs.2015.08.002 26410599 PMC4584418

[B222] Zhao Y. Dai Z. Liang Y. Yin M. Ma K. He M.. (2014). Sequence-specific inhibition of microRNA via CRISPR/CRISPRi system. Sci. Rep. 4:3943. 10.1038/srep03943 24487629 PMC3909901

[B223] Zhou X. X. Chung H. K. Lam A. J. Lin M. Z. (2012). Optical control of protein activity by fluorescent protein domains. Science 338, 810–814. 10.1126/science.1226854 23139335 PMC3702057

